# Modulating Oxygen Transfer via A‐Site Doping in LaFeO_3_ for Coke‐Resistant Chemical Looping Steam Methane Reforming

**DOI:** 10.1002/cssc.202501667

**Published:** 2025-10-08

**Authors:** Jeongin Ha, Hyeon Seok Kim, Hyunjung Kim, Yikyeom Kim, Surya Ayuati Ning Asih, Jae W. Lee

**Affiliations:** ^1^ Department of Chemical and Biomolecular Engineering Korea Advanced Institute of Science and Technology (KAIST) 291 Daehak‐Ro Daejeon 34141 Republic of Korea

**Keywords:** chemical looping steam methane reforming, coke resistance, doping, oxygen transfer, perovskite

## Abstract

Chemical looping steam methane reforming (CL‐SMR) is a promising technology for the simultaneous production of high‐purity hydrogen and syngas without the need for external gas separation units. This study evaluates a series of A‐site doped perovskite‐type oxygen carriers, La_0.8_A_0.2_FeO_3_ (A = Ca, Sr, Ba), to investigate the influence of alkaline earth metal doping on redox behavior and catalytic performance in CL‐SMR. Substituting divalent cations at the A‐site effectively promotes oxygen vacancy formation and enhances lattice oxygen transfer. Among the evaluated oxygen carriers, Sr‐doped LaFeO_3_ (La_0.8_Sr_0.2_FeO_3_) exhibits the most favorable performance. This is attributed to the optimal concentration of oxygen vacancies, which improved oxygen transfer, as confirmed by X‐ray photoelectron spectroscopy, cerimetric titration, and O_2_‐temperature programmed desorption. While undoped LaFeO_3_ (LF) exhibits the highest methane activation, its limited oxygen mobility leads to severe coke formation. Enhanced oxygen transfer in La_0.8_Sr_0.2_FeO_3_ effectively suppresses carbon deposition, while it shows the highest CO and hydrogen production. It achieves consistently high CO and H_2_ yields (6.22–6.51 and 6.48–6.69 mmol/g_cat_, respectively) and demonstrates excellent stability over 50 redox cycles.

## Graphical abstract

Promoting oxygen mobility within the oxygen carrier is essential for suppressing coke deposition during chemical looping steam methane reforming. A‐site doped LaFeO_3_ with alkaline earth metals facilitates bulk‐to‐surface oxygen transport, thereby maximizing syngas and hydrogen productivity while inhibiting carbon accumulation. Among the dopants, Sr induces the most favorable oxygen vacancy characteristics, resulting in enhanced redox performance and coke resistance.

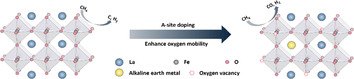

## 1 Introduction

Anthropogenic greenhouse gas emissions have significantly accelerated global warming and climate change. According to the intergovernmental panel on climate change Sixth Assessment Report, global average surface temperatures increased by 1.1 °C between 2011 and 2020, marking the most rapid period of global warming since 1970.^[^ ^1^ ^]^ To address this crisis, the Paris Agreement set goals to limit global temperature rise to well below 2 °C, with efforts to constrain it to 1.5 °C above pre‐industrial levels.^[^ ^2^, ^3^ ^]^ With many countries committing to net‐zero emissions by 2050 to achieve these goals, interest in technologies such as chemical looping is increasing due to their inherent CO_2_ capture capability without the need for additional separation units.^[^ ^4^, ^5^, ^6^ ^]^

**Figure 1:**
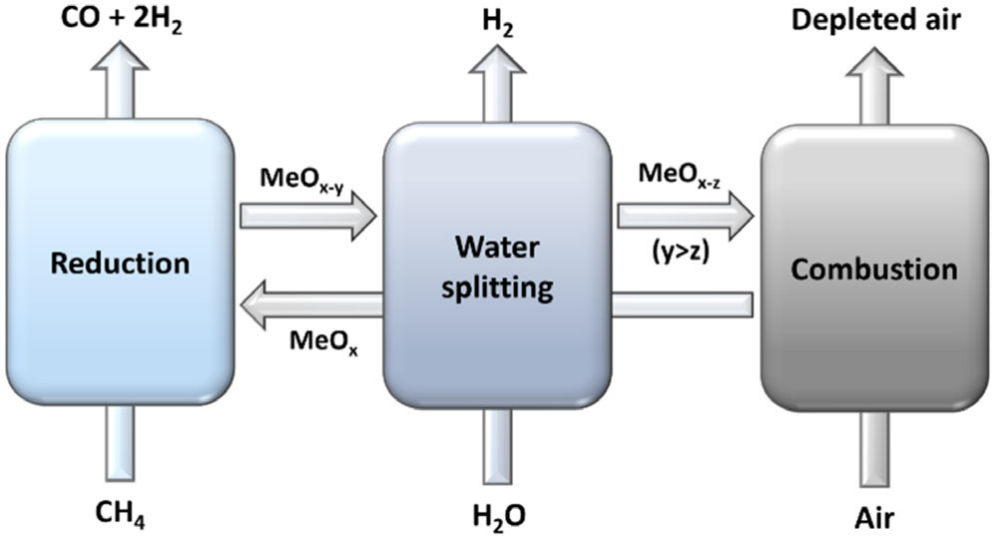
Reaction scheme of CL‐SMR.

Chemical looping steam methane reforming (CL‐SMR) is an advanced redox‐based technology that simultaneously produces syngas (CO + H_2_), which is an important feedstock for value‐added hydrocarbons, and high‐purity hydrogen.^[^ ^7^, ^8^ ^]^ As illustrated in **Figure** **1**, CL‐SMR operates through a series of cyclic redox reactions in spatially separated reactors, wherein the oxygen carrier undergoes reduction and oxidation.^[^ ^9^, ^10^ ^]^ The oxygen carrier, typically composed of metal oxides, serves dual roles as a catalyst and an oxygen source by supplying lattice oxygen to oxidize hydrocarbon fuels such as methane. In the reduction step, methane is partially oxidized by lattice oxygen in the oxygen carrier, producing syngas (Equation 1). In the subsequent water splitting step, steam is introduced and dissociated into molecular hydrogen and atomic oxygen, which reoxidizes the reduced oxygen carrier (Equation 2).^[^ ^11^ ^]^ During this step, steam also gasifies the carbonaceous deposits (coke) formed in the reduction step, producing H_2_ and CO, thereby lowering hydrogen purity. Therefore, suppressing coke formation during the methane step is essential to achieve high‐purity hydrogen production in the water splitting step. In some configurations, CL‐SMR is operated with only the methane and water splitting steps, while the subsequent air oxidation step is optional. This additional oxidation step primarily functions to fully reoxidize the oxygen carrier (Equation 3), restoring any residual oxygen deficiency, and to completely remove the coke that is not eliminated during the water splitting step.^[^ ^12^ ^]^

(1) $$\text{Reduction}:{\text{ yCH}}_{4}+{\text{MeO}}_{x}\to {\text{y(CO+2H}}_{2}{\text{)+MeO}}_{x‐y}$$

(2) $$\text{Water splitting}:\left(\text{y‐z}\right)H_{2}{\text{O+MeO}}_{x‐y}\to \left(\text{y‐z}\right)H_{2}+{\text{MeO}}_{x‐z}$$

(3) $$\text{Combustion}:0.5zO_{2}+{\text{MeO}}_{x-z}\to {\text{MeO}}_{x}$$

The performance of CL‐SMR is highly dependent on the properties of the oxygen carrier. Perovskite‐type oxides (ABO_3_),^[^ ^13^, ^14^ ^]^ which are nonstoichiometric, have gained attention due to their ability to reversibly exchange lattice oxygen without significant phase transitions and suppress metal sintering over multiple cycles.^[^ ^15^, ^16^, ^17^ ^]^ The release of lattice oxygen during the reduction step generates oxygen vacancies, which serve as active sites and enhance oxygen ion mobility within the structure.^[^ ^18^, ^19^ ^]^ Among perovskite‐type oxides, LaFeO_3_ is especially attractive due to its inherent selectivity for the partial oxidation of methane,^[^ ^20^ ^]^ an essential characteristic for efficient syngas production.^[^ ^21^ ^]^ Experimental and density functional theory (DFT) analyses revealed that LaFeO_3_ exhibits the highest reactivity and oxygen storage capacity (OSC) among AFeO_3_ (A = Ca, Sr, La) perovskites, owing to its favorable electronic structure and low energy barriers.^[^ ^22^ ^]^ Since Fe‐based perovskites exhibit relatively lower P_O2_ and a correspondingly lower oxidation potential, they promote selective partial oxidation of methane to CO and H_2_, rather than total oxidation.^[^ ^3^ ^]^ This lower oxidation potential also enhances water dissociation, enabling higher equilibrium conversion and hydrogen yield in the water splitting step of CL‐SMR.

Despite its thermodynamic advantage, pristine LaFeO_3_ suffers from relatively poor reaction kinetics, which restricts it from driving redox reactions to equilibrium.^[^ ^3^, ^23^ ^]^ To overcome this limitation, researchers have extensively explored the partial substitution of La^3+^ with divalent cations at the A‐site to enhance the formation of oxygen vacancies and oxygen migration from the bulk to the surface.^[^ ^24^, ^25^ ^]^ For example, Ca and Sr doping in LaFeO_3_ have been shown to increase the oxygen vacancy concentration significantly. This promotes lattice oxygen diffusion^[^ ^26^, ^27^, ^28^ ^]^ and enhances water adsorption on the oxygen carrier surface, in turn improving hydrogen production in the steam splitting step.^[^ ^29^ ^]^ Furthermore, enhanced oxygen transport suppresses methane decomposition, which reduces carbon deposition on the oxygen carrier surface. However, excessively enhanced oxygen diffusion rates can result in an overaccumulation of surface lattice oxygen that shifts the reaction toward methane total oxidation rather than partial oxidation. Therefore, optimizing the oxygen transfer kinetics is essential to balance reactivity, selectivity, and stability. The introduction of Ba into LaFeO_3_ significantly enhanced oxygen transport and catalytic activity, enabling efficient and stable syngas production from algae via chemical looping catalytic steam gasification, thus highlighting its promise as an oxygen carrier for biomass conversion.^[^ ^30^ ^]^ Furthermore, a recent study has demonstrated that Al‐doped La_0.6_Sr_0.4_Fe_0.8_Al_0.2_O_3−*δ*_ perovskite enables stable syngas production with high CO selectivity (≈95%) and an ideal H_2_/CO ratio of 2/1, owing to its enhanced lattice oxygen donation and coke‐free redox cycling.^[^ ^31^ ^]^

Therefore, while individual studies have reported the benefits of specific A‐site dopants such as Ca, Sr, or Ba, most of these works are limited to single‐case demonstrations without a systematic comparison. In particular, the role of different alkaline earth metals (Ca, Sr, and Ba) in governing the reactivity, oxygen mobility, and coke resistance of LaFeO_3_ under CL‐SMR conditions remains poorly understood. Building on this background, the present study investigates the impact of A‐site doping in LaFeO_3_ with alkaline earth metals (Ca, Sr, and Ba) on the performance in CL‐SMR. Specifically, the influence of substituting divalent cations at the A‐site on the formation of oxygen vacancies is examined to elucidate how these structural modifications affect the redox behavior and catalytic activity of the oxygen carriers. By comparing the effects of different dopant elements, this study aims to clarify how variations in oxygen vacancies modulate the overall performance of CL‐SMR, particularly in terms of syngas yield, hydrogen production, and coke suppression.

## 2 Results and Discussion

### 2.1 Structural Characterization of Oxygen Carriers

**Figure 2:**
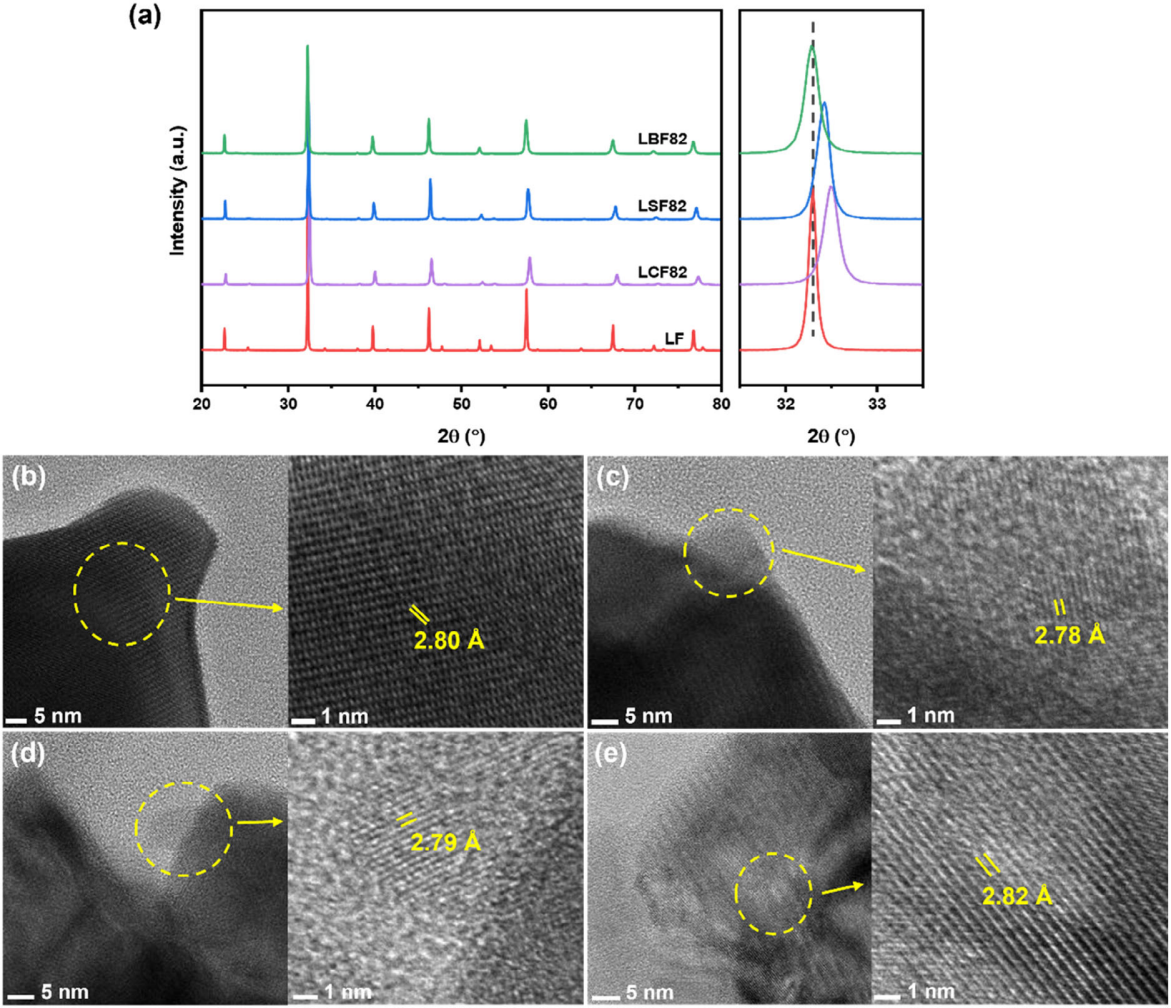
a) XRD patterns of fresh oxygen carriers; HR‐TEM images of b) LF, c) LCF82, d) LSF82, and e) LBF82.

The synthesis of samples with perovskite structures was examined using X‐ray diffraction (XRD), inductively coupled plasma optical emission spectroscopy (ICP‐OES), and transmission electron microscopy (TEM). First, XRD measurements were conducted to elucidate the crystalline structure of the synthesized oxygen carriers. As evidenced by the Rietveld refinement results (Figure S1, Supporting Information and **Table** **1**) and XRD patterns (**Figure** **2**a and S2, Supporting Information), all investigated samples exhibited diffraction patterns of an orthorhombic perovskite structure (space group Pnma, No. 62), consistent with prior reports for LaFeO_3_‐based systems.^[^ ^32^ ^]^ The absence of additional diffraction peaks indicates that Ca^2+^, Sr^2+^, and Ba^2+^ were successfully incorporated into the A‐site lattice without any secondary phases or segregation. This structural integrity was further supported by ICP‐OES, confirming the elemental compositions of the oxygen carriers (Figure S3, Supporting Information). The undoped LF sample exhibited a La: Fe atomic ratio of ≈1:1. For the doped samples (LCF82, LSF82, and LBF82), the measured La: alkaline earth metal: Fe ratios approximated the intended 0.8:0.2:1 stoichiometry, which verified that about 20% of La^3+^ was successfully substituted by divalent dopants at the A‐site. High‐angle annular dark‐field scanning TEM (HAADF‐STEM) coupled with elemental mapping (Figure S4, Supporting Information) revealed a uniform spatial distribution of all constituent elements, indicating homogeneous doping and the absence of elemental segregation. These comprehensive structural and compositional analyses collectively confirm that the homogeneously doped perovskite oxygen carriers were successfully synthesized.

**Table 1:** Refinement results of HRPD experiment for as‐synthesized perovskite.

	Lattice parameter				Cell volume [Å^3^]
	a [Å]	b [Å]	c [Å]	*α* = *β* = *γ* [°]	
LF	5.565	7.854	5.556	90	242.85
LCF82	5.523	7.805	5.533	90	238.50
LSF82	5.553	7.828	5.528	90	240.32
LBF82	5.571	7.861	5.550	90	243.06

Table 1 summarizes the lattice parameters and corresponding unit cell volumes of the synthesized perovskites, revealing an expansion with increasing dopant ionic radius. Specifically, both the lattice constants and cell volume increase progressively in the order of LCF82 < LSF82 < LF < LBF82, which is consistent with the established ionic radius of A‐site cation, Ca^2+^ (0.99 Å) < Sr^2+^ (1.12 Å) < La^3+^ (1.16 Å) < Ba^2+^ (1.35 Å).^[^ ^25^ ^]^ The XRD patterns corroborate this lattice expansion trend (Figure 2a). The main diffraction peak near 2*θ* = 32°–33°, which is the peak for the (121) plane, shifts toward lower angles as the unit cell volume increases, and vice versa. These peak shifts indicate increased interplanar spacing (d‐spacing), attributable to enlargement of the lattice parameter.^[^ ^33^ ^]^ Consequently, the d‐spacing for the (121) plane follows the order LBF82 > LF > LSF82 > LCF82. This is further confirmed by HR‐TEM, as shown in Figure 2b–e, where the measured d‐spacings of the (121) plane are 2.80 Å for LF, 2.78 Å for LCF82, 2.79 Å for LSF82, and 2.82 Å for LBF82.^[^ ^34^ ^]^ The excellent agreement between the HR‐TEM and XRD results reinforces the successful incorporation of alkaline‐earth dopants into the A‐site lattice, resulting in systematic structural modifications in line with the dopant size.

Similarly, the main diffraction peak located at 2*θ* = 32°–33° consistently shifts toward higher angles with increasing Ca or Sr doping levels (Figure S2a,b, Supporting Information).^[^ ^35^ ^]^ This peak shift also indicates a decrease in d‐spacing, which is attributed to the partial substitution of La^3+^ by smaller Ca^2+^ or Sr^2+^ ions within the A‐site of the perovskite lattice. The effect of lattice contraction can also be explained by the ionic charge. Incorporating divalent Ca^2+^ or Sr^2+^ in a trivalent La^3+^ site introduces a charge imbalance to the lattice. To maintain charge neutrality, the system compensates either through the formation of oxygen vacancies or via the oxidation of Fe^3+^ to Fe^4+^. Since Fe^4+^ (0.585 Å) possesses a smaller ionic radius than Fe^3+^ (0.645 Å), lattice contraction occurs, further contributing to the peak shift toward higher diffraction angles.^[^ ^28^ ^]^ However, in the case of Ba‐doped samples, the diffraction peaks shift toward lower angles as the Ba doping ratio increases (Figure S2c, Supporting Information). This shift is attributed to the larger ionic radius of Ba^2+^ compared to La^3+^, which results in an expansion of the lattice and an increase in the d‐spacing. Overall, these results confirm that all samples were successfully synthesized as homogeneous orthorhombic perovskite structures without detectable secondary phases, indicating that the dopants were effectively incorporated at the A‐site of LaFeO_3_ as intended.

### 2.2 Evaluation of Oxygen Vacancies and Oxygen Content

**Figure 3:**
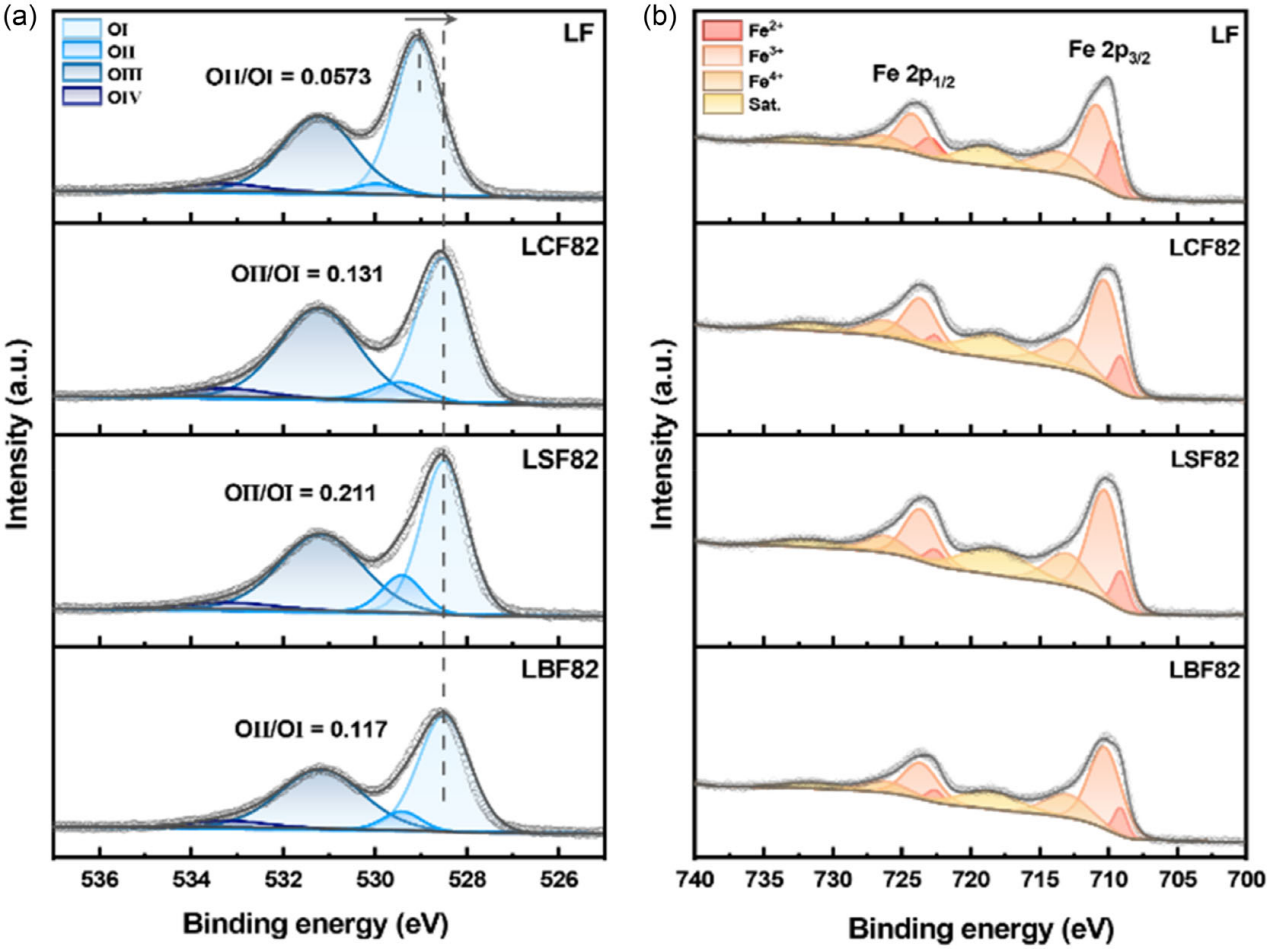
XPS spectra of a) O *1s* and b) Fe *2* *p* for LF, LCF82, LSF82, and LBF82.

To investigate oxygen vacancies and oxygen contents in the oxygen carriers, X‐ray photoelectron spectroscopy (XPS) and cerimetric titration were performed. A O *1* *s* XPS analysis was conducted to investigate the concentration of surface oxygen vacancies. The surface oxygen species were characterized via O *1s* XPS spectra (**Figure** **3**a and **Table** **2**), which were deconvoluted into four distinct components: OI (lattice oxygen, O^2−^), OII (surface adsorbed oxygen or oxygen vacancy, O_2_^2−^/O^−^), OIII (carbonate or hydroxyl species, CO_3_^2−^/OH^−^), and OIV (molecular water, H_2_O).^[^ ^36^, ^37^ ^]^ Notably, the binding energy of the undoped LF sample was higher than that observed in the doped samples (LCF82, LSF82, and LBF82). This indicated lower electron density and, consequently, a higher proportion of electrophilic oxygen species in LF, which may favor redox activity in the undoped sample.^[^ ^38^ ^]^ Given that surface‐adsorbed oxygen species are generally correlated with oxygen vacancy concentrations, the OII/OI ratio serves as a proxy for oxygen vacancy concentration.^[^ ^25^, ^39^, ^40^ ^]^ The calculated OII/OI ratios, as presented in Table 2, provide insight into the relative concentration of surface oxygen vacancies among the samples. LSF82 exhibited the highest OII/OI ratio (0.211), followed by LCF82 (0.131), LBF82 (0.117), and LF (0.0573), indicating that LSF82 possesses the highest concentration of surface oxygen vacancies. Since a greater concentration of oxygen vacancies will enhance lattice oxygen mobility and facilitate oxygen exchange processes at the surface, LSF82 is expected to exhibit superior redox performance compared to the other samples.

**Table 2:** Atomic ratio of oxygen species and OII/OI determined by O *1s* XPS.

at%	OI (O^2−^)	OII (O_2_^2−^, O^−^)	OIII (CO_3_^2−^, OH^−^)	OIV (H_2_O)	OII/OI
LF	53.62	3.07	39.35	3.96	0.0573
LCF82	45.70	5.98	44.39	3.93	0.131
LSF82	45.66	9.64	41.01	3.69	0.211
LBF82	49.44	5.77	40.66	4.13	0.117

Further evidence for the relative concentration of oxygen content and oxygen vacancies was obtained from a Fe *2p* XPS analysis. As shown in Figure 3b, the Fe *2p* spectra of all samples display the expected spin‐orbit doublets corresponding to Fe *2p*_1/2_ and Fe *2p*_3/2_, and each doublet is deconvoluted into Fe^2+^, Fe^3+^, Fe^4+^, and satellite peaks.^[^ ^39^ ^]^ The relative abundance of these oxidation states and the average cationic charge of Fe species are shown in **Table** **3**. Notably, the undoped LF sample exhibits a higher proportion of Fe^2+^, whereas the doped samples (LCF82, LSF82, and LBF82) show increases of Fe^3+^ and Fe^4+^ species due to charge compensation.^[^ ^29^, ^37^, ^39^ ^]^ The relative concentration of surface oxygen vacancies in the oxygen carriers is closely related to the total cationic charge. As summarized in Table 3, the average oxidation state of Fe, determined from Fe *2* *p* XPS fitting, was combined with the A‐site cationic charge to obtain the total cationic charge to estimate the oxygen content and, subsequently, the oxygen vacancy concentration (*δ*).^[^ ^37^ ^]^ The calculated oxygen contents (3‐*δ*) decrease in the order LF > LBF82, LCF82 > LSF82, indicating a progressive increase in the oxygen vacancy concentration across the series. The corresponding *δ* values were negative for LF, 0.014 for LCF82, 0.024 for LSF82, and 0.013 for LBF82. Among the samples, LSF82 exhibited the highest concentration of oxygen vacancies, followed by LCF82, LBF82, and LF. This trend is consistent with the OII/OI ratio obtained from O *1* *s* XPS analysis and further confirms that LSF82 possesses the highest concentration of surface oxygen vacancies. Collectively, the results from both the O *1* *s* and Fe *2* *p* XPS analyses corroborate that LSF82 has the highest and LF has the lowest relative surface oxygen vacancy concentration among the tested samples.

**Table 3:** Atomic ratio of iron species determined by Fe *2p* XPS and oxygen contents.

	Fe^2+^ a)	Fe^3+^ a)	Fe^4+^ a)	Average charge of Fe speciesb)	A‐site charge	Total cationic chargeb)	Oxygen contents (3−δ)b)	Oxygen contents (3−δ)c)
LF	18.55	56.50	29.96	3.06	3.00	6.06	3.03	3.00
LCF82	10.68	61.64	27.71	3.17	2.80	5.97	2.99	2.99
LSF82	10.32	64.12	25.55	3.15	2.80	5.95	2.98	2.97
LBF82	9.24	64.20	26.56	3.17	2.80	5.97	2.99	2.98

a) Atomic ratio (at%) of iron species fitted by Fe *2* *p* XPS.

b) Calculated values from Fe *2* *p* XPS fitting results.

c) Calculated values from cerimetric titration.

While an XPS analysis was used to estimate the concentration of surface oxygen vacancies, cerimetric titration was carried out to determine the total oxygen content within the oxygen carrier experimentally. This method quantifies the Fe^2+^ or Fe^4+^ content in the perovskite structure, from which the overall oxygen content can be calculated. As summarized in Table 3 (Figure S5, Supporting Information), the oxygen content increases in the order of LSF82 < LBF82 < LCF82 < LF. Given the inverse relationship between oxygen content and oxygen vacancy concentration, the corresponding oxygen vacancy concentrations (*δ*) follow the reverse trend: 0.027 for LSF82, 0.019 for LBF82, 0.014 for LCF82, and zero for LF. These total oxygen vacancy values show a consistent trend with the surface oxygen vacancy concentrations estimated by XPS, further supporting that LSF82 possesses the highest concentration of oxygen vacancies, while LF has the lowest. The elevated vacancy concentration in LSF82 is expected to enhance oxygen mobility within the lattice, offering a kinetic advantage in redox reactions.

### 2.3 Estimation of Oxygen Mobility and Redox Activity Through Temperature‐Programmed Experiments

**Figure 4:**
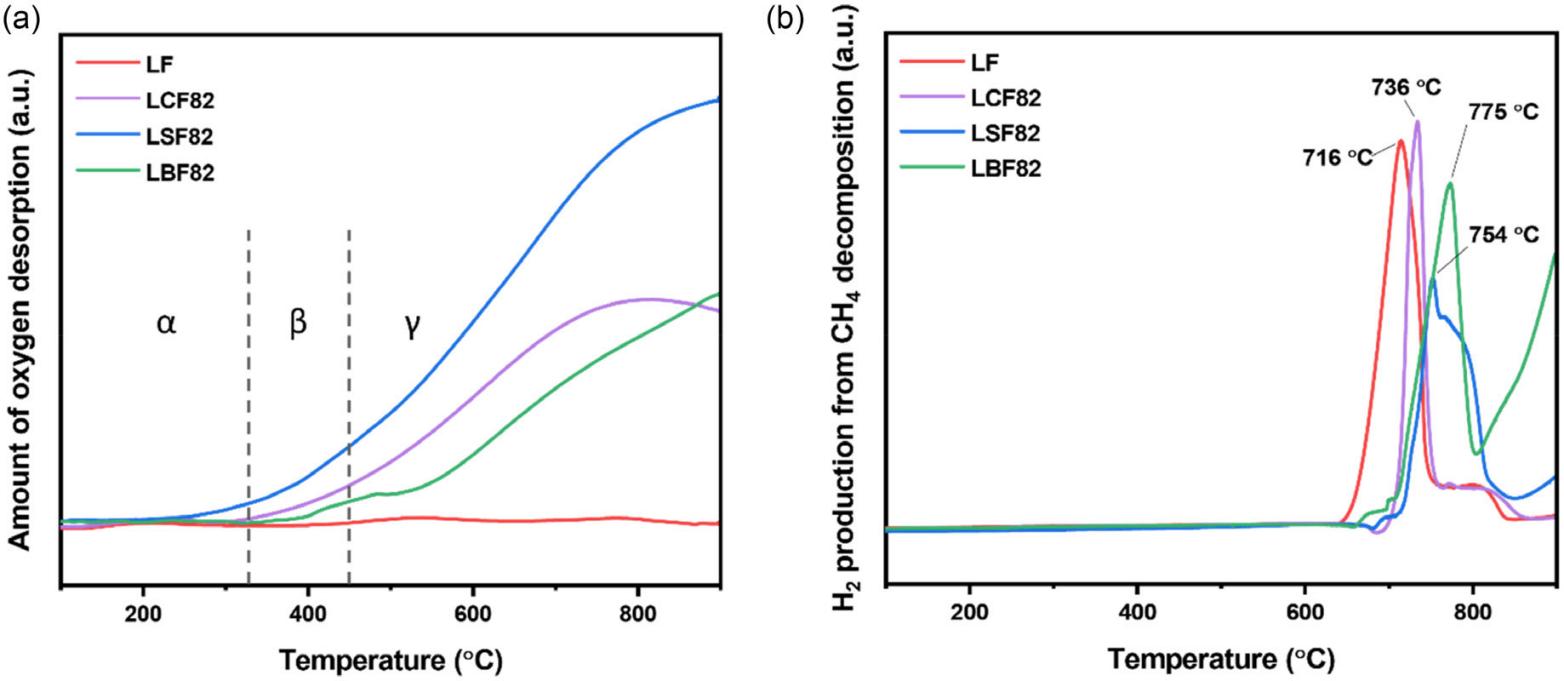
Results of a) O_2_‐TPD and b) CH_4_‐TPSR for LF, LCF82, LSF82, and LBF82.

To elucidate the redox characteristics of the synthesized oxygen carriers, temperature‐programmed analyses were conducted. Experimentally, O_2_‐temperature programmed desorption (O_2_‐TPD) evaluates the effect of oxygen vacancies on oxygen mobility in perovskite materials. The oxygen desorption behavior of the oxygen carriers was examined to gain deeper insight into their oxygen transfer from the bulk to the surface. As illustrated in **Figure** **4**a, the desorption profiles can be divided into three distinct temperature regions, each corresponding to different types of oxygen species.^[^ ^25^, ^41^ ^]^ The α‐region (150–320 °C) is associated with the release of physically adsorbed molecular oxygen (O_2_) and weakly bound surface species such as O_2_^−^ or O_2_^2−^, which are loosely adsorbed to surface oxygen vacancies. The β‐region (320‐450 °C) represents the desorption of more strongly chemisorbed oxygen species on the surface (O_2_^−^ or O_2_^2−^) and sub‐surface oxygen (O^−^). Lastly, the γ‐region (>450 °C) is attributed to the desorption of lattice oxygen (O^2−^) from the perovskite bulk. Notably, the *β* and *γ* regions are particularly related to oxygen transfer from the bulk to the surface. The calculated total amount of oxygen released from O_2_‐TPD follows the order of LSF82 > LCF82 > LBF82 > LF (Table S1, Supporting Information). A greater total oxygen release not only reflects the rapid desorption of surface oxygen but also indicates the facile migration of lattice oxygen toward the surface. This demonstrates superior oxygen mobility of LSF82, which is essential for maintaining high reactivity during CL‐SMR. Among the samples, LSF82 exhibited the highest oxygen desorption, particularly in the β and γ regions, followed by LCF82, LBF82, and LF. This observation suggests that LSF82 allows more effective oxygen migration from the bulk to the surface due to enhanced oxygen vacancy concentration, which is a critical factor for redox‐based CL‐SMR. In contrast, LCF82 and LBF82, which have comparatively lower oxygen vacancy concentrations, exhibited reduced oxygen mobility, as evidenced by the diminished O_2_ desorption amount in these regions. Furthermore, LF, which possesses a negligible oxygen vacancy concentration, displayed almost no oxygen desorption across the entire temperature range. Compared with LF, LCF82, and LBF82, the superior oxygen transfer capability of LSF82 is due to its higher concentration of oxygen vacancies, as confirmed by XPS and cerimetric titration results. These results suggest that the superior oxygen exchange kinetics of LSF82 may underlie its enhanced redox performance and resistance to carbon deposition during CL‐SMR, which will be discussed in detail in the subsequent sections of the CL‐SMR experiment.

From the results of XPS and temperature‐programmed experiments, LSF82 exhibits the highest concentration of oxygen vacancies, which is beneficial for enhancing oxygen transfer and redox performance. A significant variation in the oxygen vacancy concentration is observed despite doping with divalent cations having the same oxidation state (Ca^2+^, Sr^2+^, or Ba^2+^) at an identical molar ratio. This discrepancy arises from differences in solution and oxygen vacancy binding energy, as reported in previous computational and experimental studies.^[^ ^42^, ^43^ ^]^ The solution energy represents the total energy involved in replacing La^3+^ with a lower‐valent cation and simultaneously generating oxygen vacancies to maintain charge neutrality.^[^ ^44^ ^]^ Therefore, a lower solution energy indicates that the lower‐valent dopant is more readily incorporated into the LaFeO_3_ lattice and more likely to induce the formation of oxygen vacancies. DFT calculations by Taylor et al. demonstrated that Sr‐doped LaFeO_3_ has the lowest solution energy among the three dopants (Ca, Sr, and Ba), indicating that Sr^2+^ is more readily incorporated into the perovskite lattice and generates a greater extent of oxygen vacancies.^[^ ^43^ ^]^ In addition, among the Ca‐, Sr‐, and Ba‐doped LaFeO_3_‐based perovskites, the Sr‐doped sample exhibits the most positive binding energy between the dopant and oxygen vacancies. A more positive binding energy indicates a weaker interaction between the dopant and the oxygen, which allows easier migration of oxygen ions into the vacancy sites. This energetically different behavior can be rationalized based on the ionic radius: the ionic radius of Sr^2+^ (1.12 Å) closely approximates that of La^3+^ (1.16 Å), resulting in minimal lattice distortion upon substitution.^[^ ^44^ ^]^ In contrast, a greater mismatch in ionic size, as in the cases of Ca^2+^ (0.99 Å) and Ba^2+^ (1.35 Å), induces more significant lattice strain. This not only increases the solution energy but also strengthens dopant‐oxygen binding, thereby suppressing oxygen vacancy formation and mobility.^[^ ^43^, ^45^ ^]^ Consequently, although all three dopants have the same oxidation state, Sr‐doped LaFeO_3_ achieves a higher oxygen vacancy concentration due to its optimal ionic radius, which facilitates dopant incorporation and promotes oxygen ion transport. Hence, LSF82 is more advantageous for redox reactions in CL‐SMR due to its facile oxygen transfer.

In addition to the oxygen vacancy concentration and oxygen mobility, the reactivity of methane on the surface of the oxygen carrier is also a critical factor for CL‐SMR performance. The catalytic activity of the synthesized perovskite oxygen carriers toward methane activation was investigated via CH_4_‐temperature programmed surface reaction (CH_4_‐TPSR).^[^ ^36^, ^46^ ^]^ Figure 4b illustrates the hydrogen evolution profile derived exclusively from methane decomposition. Since hydrogen can be produced from both methane partial oxidation (Equation 5) and methane decomposition (Equation 6), the contribution of partial oxidation was excluded. During the partial oxidation, one mole of CH_4_ produces one mole of CO and two moles of H_2_. Hence, the amount of H_2_ attributable to the partial oxidation can be estimated as twice the amount of CO formed. By subtracting this amount from the total amount of hydrogen produced, the hydrogen originating from the methane decomposition was calculated, and the result was illustrated in Figure 4b. The complete effluent gas profiles, including CH_4_, CO, CO_2_, and H_2_, are available in (Figure S6, Supporting Information), where the peak temperature for CO production is lowest for LF, followed by LCF82, LSF82, and LBF82. A similar trend is observed in Figure 4b, where distinct H_2_ formation peaks corresponding to methane decomposition occur at 716 °C for LF, 736 °C for LCF82, 754 °C for LSF82, and 775 °C for LBF82, respectively. Since lower peak temperatures indicate methane activation at lower thermal energy, this trend suggests that methane reactivity on the oxygen carrier surface follows the order LF > LCF82 > LSF82 > LBF82.^[^ ^46^ ^]^ The highest methane reactivity observed for LF may be associated with its surface oxygen species, which exhibit relatively lower electron density, as inferred from the higher binding energy in the O *1s* XPS spectra. While binding energy alone does not definitively quantify oxidizing ability, this trend aligns with the enhanced CH_4_ activation observed in TPSR, suggesting that the electrophilic surface oxygen in LF may play a more active role in methane activation. While the high reactivity of LF is advantageous for achieving elevated CH_4_ conversion, it also implies an increased risk of methane decomposition on the LF surface, which would result in carbon deposition on the oxygen carrier. Because methane decomposition initiates on LF at lower temperatures, at identical reaction temperature, LF is more susceptible to undergo methane decomposition than LCF82, LSF82, or LBF82. In the context of CL‐SMR, such excessive methane decomposition during the reduction step leads to coke formation on the oxygen carrier surface, which adversely affects the catalyst stability and reduces H_2_ purity during the subsequent water splitting step. Therefore, compared to the other samples, LF is expected to generate a greater amount of coke during the methane step in CL‐SMR, which would negatively impact the production of high‐purity hydrogen in the subsequent steam step.

In addition, the OSC can be calculated from the amounts of CO_2_ and CO produced during CH_4_‐TPSR. The OSC determined from CH_4_‐TPSR is presented in the supporting information (Table S1, Supporting Information), together with the ratio of actual to the theoretical oxygen release.^[^ ^47^ ^]^ A comparison with literature values shows that the OSC values in this study are higher than those previously reported for other perovskites (Table S2, Supporting Information). The results show that the fraction of oxygen released with respect to the theoretical value follows the order of LSF82 > LBF82 > LF > LCF82. This suggests that in practical CL‐SMR processes, lattice oxygen in LSF82 can more rapidly migrate to the surface and react with methane to produce syngas. In contrast, for LF and LCF82, methane decomposition occurs earlier than in LSF82 or LBF82, leading to more severe coke deposition on the surface. Such coke formation hinders the migration of lattice oxygen to the surface, leading to a smaller amount of oxygen released compared with the theoretical value. Therefore, under actual redox conditions, LSF82 can release and uptake lattice oxygen more effectively than the other samples, thereby promoting syngas generation while suppressing coke deposition in the CL‐SMR process. Overall, the influence of methane reactivity and oxygen mobility on CL‐SMR performance is further validated and discussed in the following sections.

### 2.4 Optimal Doping Ratio and Reaction Temperature for CL‐SMR Experiments

Before the cyclic experiments, preliminary tests were performed to determine the optimal reaction temperature and doping ratio (see the “Extended experimental section” in Supporting Information). To determine the optimal doping level, LaFeO_3_‐based perovskites with A‐site doping levels (Ca, Sr, or Ba) of 10%, 20%, and 30% were tested at 750 °C (Figure S7a–c, Supporting Information). Among these, the 20% doped samples (LCF82, LSF82, and LBF82) exhibited the highest overall performance during the CL‐SMR experiments. Specifically, within the LCF series, LCF82 showed the best performance, exhibiting the highest CO selectivity, the lowest coke formation during the methane step, and the highest H_2_ production during the water splitting step. Likewise, LSF82 displayed superior performance among the LSF series, with the highest CO selectivity and H_2_ yield. Although coke formation in LSF82 was also the highest, it is comparable across the LSF series, showing relatively small differences. In the case of the LBF series, LBF82 showed the highest CO selectivity and lowest coke formation and the second‐highest H_2_ yield. According to previous studies, increasing the doping ratio introduces a larger charge imbalance in the perovskite lattice, which leads to a higher concentration of oxygen vacancies.^[^ ^21^ ^]^ This promotes more rapid oxygen release to the surface, favoring the total oxidation of methane rather than partial oxidation. Conversely, a lower doping ratio results in a smaller charge imbalance and reduced oxygen vacancy concentration, which slows the migration of lattice oxygen to the surface.^[^ ^37^ ^]^ As a result, partial oxidation is suppressed and methane decomposition is promoted, leading to increased coke deposition. These findings indicate that 20% A‐site doping is optimal for enhancing partial oxidation of methane to syngas while simultaneously minimizing coke formation during CL‐SMR.

Thereafter, LSF82 was tested at 700, 750, and 800 °C to identify the optimal reaction temperature (Figure S7d, Supporting Information), and the highest CO selectivity and H_2_ yields were achieved at 800 °C. Notably, the amount of coke formed remained relatively consistent across all tested temperatures. This suggests that 800 °C is the most effective condition for maximizing syngas and hydrogen production without promoting excessive carbon formation. Therefore, 800 °C was selected as the optimal reaction temperature for further cyclic CL‐SMR experiments with a 20% doping ratio.

### 2.5 Performance During the Reduction Step in CL‐SMR Experiments

**Figure 5:**
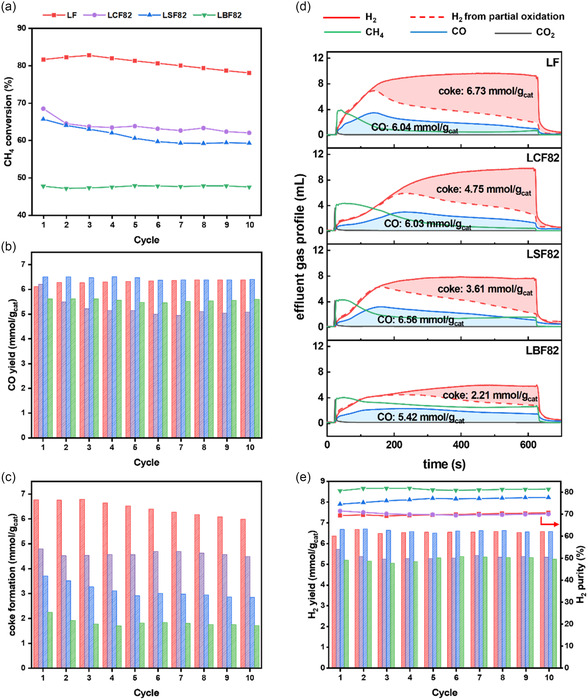
CL‐SMR performance for LF, LCF82, LSF82, and LBF82: a) CH_4_ conversion, b) CO yield, and c) estimated coke deposition during the reduction step, d) real‐time effluent gas profile during the reduction step, and e) H_2_ yield and purity from the water splitting step.

With the optimal doping ratio and reaction temperature established, cyclic CL‐SMR experiments were carried out at 800 °C using the oxygen carriers LF, LCF82, LSF82, and LBF82. The performance of the oxygen carriers in the CL‐SMR reactions was primarily evaluated based on the methane conversion, and CO and coke yields during the methane reduction step. In **Figure** **5**a, the methane conversion followed the order LF > LCF82 > LSF82 > LBF82 at the first cycle of the reduction step. This trend is consistent with the CH_4_ activation tendency observed in CH_4_‐TPSR (Figure 4b), where LF exhibited the highest methane reactivity as indicated by its lowest methane decomposition peak temperature, followed by LCF82, LSF82, and LBF82. Quantitatively, the methane conversion of the first cycle was 81.6% for LF, 68.4% for LCF82, 65.6% for LSF82, and 47.6% for LBF82. The relatively low reactivity of LBF82 toward methane resulted in significantly lower CH_4_ conversion, and consequently, minimal CO yield and coke formation, as shown in Figure 5b and c. Both the CO yield and coke deposition behavior reflect the combined influence of methane activation and oxygen mobility. As previously discussed in Section 2.2, 2.3, the oxygen vacancy concentration, and hence the oxygen mobility, follows the order: LSF82 > LCF82 > LBF82 > LF. This increasing mobility correlates with the CO yield trend among the doped samples: LSF82 exhibited the highest CO yield in the first cycle (6.73 mmol/g_cat_), followed by LCF82 (6.03 mmol/g_cat_) and LBF82 (5.42 mmol/g_cat_). Interestingly, although LF possesses the lowest oxygen vacancy concentration and correspondingly the lowest oxygen mobility, it showed a comparable CO yield (6.04 mmol/g_cat_) to that of LCF82. This can be primarily attributed to its superior methane activation, as evidenced by CH_4_‐TPSR. The high reactivity of methane on LF likely compensates for its limited oxygen transport capacity and results in comparable CO production.

Although the methane conversion of LF is ≈20% higher than that of LSF82, its CO yield is comparable to or even slightly lower than that of LSF82, with a value of 6.11–6.39 mmol/g_cat_ for LF and 6.37‐6.50 mmol/g_cat_ for LSF82. This discrepancy suggests that a substantial portion of methane conversion over LF proceeds via nonoxidative decomposition, resulting in the formation of solid carbon and hydrogen, rather than selective partial oxidation to CO. This interpretation is supported by the coke yield data presented in Figure 5c, which shows that LF accumulates 5.99–6.76 mmol/g_cat_ of coke, nearly double the amount of coke produced with LSF82 (2.85–3.70 mmol/g_cat_). Further insights are provided by the real‐time effluent gas profile during the methane step of the first cycle (Figure 5d).^[^ ^48^, ^49^ ^]^ In the initial stage (0–20 s), methane undergoes total oxidation (Equation 4), producing CO_2_ and H_2_O.^[^ ^17^, ^50^ ^]^ This is followed by a regime dominated by partial oxidation (Equation 5), lasting up to approximately three minutes, where CO and H_2_ are generated. Beyond this point, both partial oxidation and methane decomposition (Equation 6) occur concurrently.^[^ ^51^, ^52^ ^]^ The simultaneous progression of these reactions is evidenced by the observation that the amount of produced H_2_ is more than twofold the amount of CO, which would be the expected ratio if only partial oxidation occurred. This indicates that methane decomposition begins even before all lattice oxygen is consumed. Furthermore, the examination of coke formation with respect to the amount of oxygen released during the methane step indicates that coke begins to form in all samples before the complete release of lattice oxygen (Figure S8, Supporting Information). However, in LSF82, the onset of carbon formation is delayed, and the overall coke deposition follows the order of LCF82 > LF > LBF82 > LSF82. These results confirm that, regardless of the reaction time during the methane step, LSF82 most effectively suppresses the coke deposition.

(4) $$\text{Total oxidation}:{\text{ CH}}_{4}+4\left[O\right]\to {\text{CO}}_{2}+2H_{2}O$$

(5) $$\text{Partial oxidation}:{\text{ CH}}_{4}+\left[O\right]\to \text{CO}+2H_{2}$$

(6) $$\text{Methane decomposition}:{\text{ CH}}_{4}\to C\left(s\right)+2H_{2}$$

The methane decomposition before complete lattice oxygen consumption suggests that the rate of oxygen diffusion from the bulk to the surface is insufficient to match the rate of methane cleavage. Consequently, methane adsorbed on the oxygen carrier is more likely to undergo decomposition in the absence of sufficient surface oxygen, leading to severe coke accumulation and reduced CO yield. This kinetic mismatch between CH_4_ activation and oxygen transfer is especially pronounced in LF, where the low oxygen vacancy concentration limits the oxygen transfer. In contrast, LSF82 benefits from an elevated oxygen vacancy concentration, which enhances oxygen diffusion to the surface and provides more efficient oxidation of surface methane. This suppresses coke formation and allows for higher CO yields, despite lower overall methane conversion compared to LF. Although LCF82 also demonstrates reduced coke formation and CO yields comparable to LF, its oxygen mobility is inferior to that of LSF82, resulting in less effective suppression of methane decomposition. LBF82, while exhibiting the lowest coke yield among all samples, also shows the lowest CO production. This is attributed to both its limited oxygen mobility and its intrinsically lower reactivity toward methane, as previously discussed. Thus, Sr doping in LaFeO_3_ significantly promotes the formation of oxygen vacancies and thereby enhances oxygen mobility. This enables more effective surface oxidation of methane, improving syngas yield while substantially mitigating coke formation compared to other samples.

Following the first cycle, a decline in methane conversion was observed for LF, LCF82, and LSF82. As illustrated in Figure 5b,c, which present the CO yield and coke formation during the methane step, both LF and LSF82 maintained relatively stable CO yields between the 1st and 10th cycles, despite a noticeable reduction in the amount of coke generated. This indicates that the observed decrease in the methane conversion for LF and LSF82 is primarily attributed to the suppression of methane decomposition rather than a loss of catalytic activity for partial oxidation. In contrast, LCF82 exhibited a pronounced decline in both methane conversion and CO yield over successive cycles. Specifically, the methane conversion decreased from 68.4% in the first cycle to 62.0% in the tenth cycle, while the CO yield dropped from 6.20 to 5.08 mmol/g_cat_. This parallel decrease suggests a degradation in the oxygen mobility as the redox cycling progressed. This inference is corroborated by the O_2_‐TPD analysis of fresh and spent samples after 10 cycles (Figure S9, Supporting Information). While LF, LSF82, and LBF82 exhibited nearly identical oxygen desorption behavior before and after CL‐SMR cycling, LCF82 showed a marked reduction in oxygen desorption, particularly in the *β*‐region (320–450 °C) and *γ*‐region (>450 °C).^[^ ^25^, ^41^ ^]^ This decline reflects a significant reduction in the migration of lattice oxygen from the bulk to the surface and confirms that the oxygen mobility in LCF82 deteriorates with repeated redox operation.^[^ ^53^ ^]^

The distinct performance degradation observed exclusively in the LCF82 sample is attributed to its inherent structural instability.^[^ ^54^ ^]^ The XRD and Fe *2* *p* XPS results of the samples reduced in methane at 800 °C for 10 min are given in the supporting information (Figure S10 and S11a, Supporting Information). The XRD patterns reveal that a part of the perovskite was transformed into La_2_O_3_ and metallic Fe, which is consistent with the presence of Fe^0^ species observed in the Fe *2* *p* XPS spectra. In addition, compared with the fresh sample, the proportions of Fe^4+^ and Fe^3+^ decreased while the proportion of Fe^2+^ increased after reduction (Table S3, Supporting Information). These results indicate that during the methane step of CL‐SMR, lattice oxygen in the perovskite is released to react with methane, thereby reducing the Fe species from higher oxidation states (Fe^4+^ or Fe^3+^) to lower oxidation states (Fe^2+^ or Fe^0^). The XRD analysis, followed by Rietveld refinement of the spent samples after 10 CL‐SMR cycles (Figure S12 and Table S4, Supporting Information), confirmed that all samples maintained their original orthorhombic perovskite structure observed in fresh samples without the formation of secondary phases. This indicates that the reduced perovskite during the methane step was well restored during the oxidation process, returning to its original orthorhombic phase. Despite this structural recovery, lattice parameters and unit cell volume increased in all spent samples relative to their fresh counterparts. Among the samples, LCF82 exhibited the largest increase in unit cell volume upon redox cycling, suggesting a lower structural recovery under operating conditions compared to the other samples. The fitted O *1* *s* XPS spectra of the spent samples after 10 CL‐SMR cycles, together with the atomic ratios of each oxygen species, are presented in the supporting information (Figure S11b and Table S5, Supporting Information). The comparison of the OII/OI ratio in the spent samples (Table S5, Supporting Information) with that of the fresh samples (Table 2) reveals a decrease in all samples except LSF82, with the most pronounced decrease observed for LCF82. This indicates that LCF82 lost the largest amount of oxygen vacancies after cycling, whereas LSF82 retained them most effectively.

This structural susceptibility is likely due to the large ionic radius mismatch between the dopant, Ca^2+^, and the host A‐site cation, La^3+^. This is further supported by the calculated Goldschmidt tolerance factor showing the largest deviation from the ideal cubic structure, whose value is 1 (Figure S13, Supporting Information).^[^ ^55^ ^]^ The tolerance factor serves as a measure of geometric distortion from the ideal perovskite structure.^[^ ^17^ ^]^ LCF82 exhibits the tolerance factor furthest from the ideal value of 1, supporting that it has a greater degree of structural distortion. As a result, LCF82 undergoes a significant expansion of the unit cell during cycling. This likely hinders the diffusion of lattice oxygen, which reduces the CO yield and increases the propensity for methane decomposition. These findings highlight the critical role of dopant selection in maintaining structural stability and sustaining redox performance in CL‐SMR applications.

### 2.6 Performance During the Water‐Splitting Step in CL‐SMR Experiments

In the subsequent water splitting step, the performance of the oxygen carriers in the CL‐SMR experiments was investigated based on the H_2_ yield and purity. Figure 5e illustrates the hydrogen production performance of the oxygen carriers during the water splitting step. In this stage, steam reacts at the surface of the reduced oxygen carrier, dissociating into molecular hydrogen and atomic oxygen. The resulting atomic oxygen is subsequently incorporated into the lattice, reoxidizing the oxygen carrier (Equation (2)). Therefore, the extent of hydrogen generation during the steam step is closely tied to the degree of lattice oxygen consumption during the preceding methane reduction step. Greater oxygen extraction from the lattice allows for more reoxidation, and consequently, higher hydrogen production during the steam reaction. Thus, the H_2_ yield during the water splitting step mirrors the trend observed for the CO yield during the methane step. The hydrogen yields follow the order LSF82 (6.55‐6.69 mmol/g_cat_) > LF (6.35–6.57 mmol/g_cat_) > LCF82 (5.23–5.71 mmol/g_cat_) > LBF82 (5.04‐5.35 mmol/g_cat_).

In parallel with lattice oxygen recovery during the steam splitting step, a competing side reaction occurs wherein residual coke formed during the methane step is gasified by steam, generating additional CO and H_2_ (Equation (7)), which decreases the H_2_ purity.^[^ ^56^, ^57^ ^]^ Although the H_2_ yields of LSF82 and LF are comparable, the substantial coke deposition observed for LF during the methane step leads to increased CO formation during the steam step, ultimately reducing the hydrogen purity. The amount of coke formed during methane reduction follows the order LF > LCF82 > LSF82 > LBF82. Accordingly, the hydrogen purity exhibits the reverse trend, with LF producing the least pure hydrogen and LBF82 the highest. Although LBF82 exhibited the highest hydrogen purity, its hydrogen yield is the lowest among the samples. This is attributable to its limited oxygen mobility and low methane reactivity, which results in minimal lattice oxygen consumption during the reduction step and thus minimal regeneration during steam exposure. Therefore, among all the samples, LSF82 achieves the highest hydrogen production with relatively high purity.
(7)

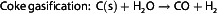




In conclusion, LSF82 provides both the highest CO yield and relatively high‐purity hydrogen yield while effectively suppressing coke deposition on the catalyst surface. This superior performance is ascribed to the increased oxygen vacancy concentration induced by Sr doping in the A‐site, which enhances oxygen transfer and promotes efficient CH_4_ partial oxidation. The improved oxygen mobility mitigates coke formation and enables rapid lattice regeneration across the redox cycles. Furthermore, the similarity of the ionic radius between Sr^2+^ and the host cation La^3+^ contributes to structural stability, allowing LSF82 to maintain consistent CO and H_2_ yields over 10 cycles. Thus, LSF82 emerges as a highly promising oxygen carrier for CL‐SMR, offering a favorable balance of activity, selectivity, and durability.

### 2.7 Extended CL‐SMR Experiments

**Figure 6:**
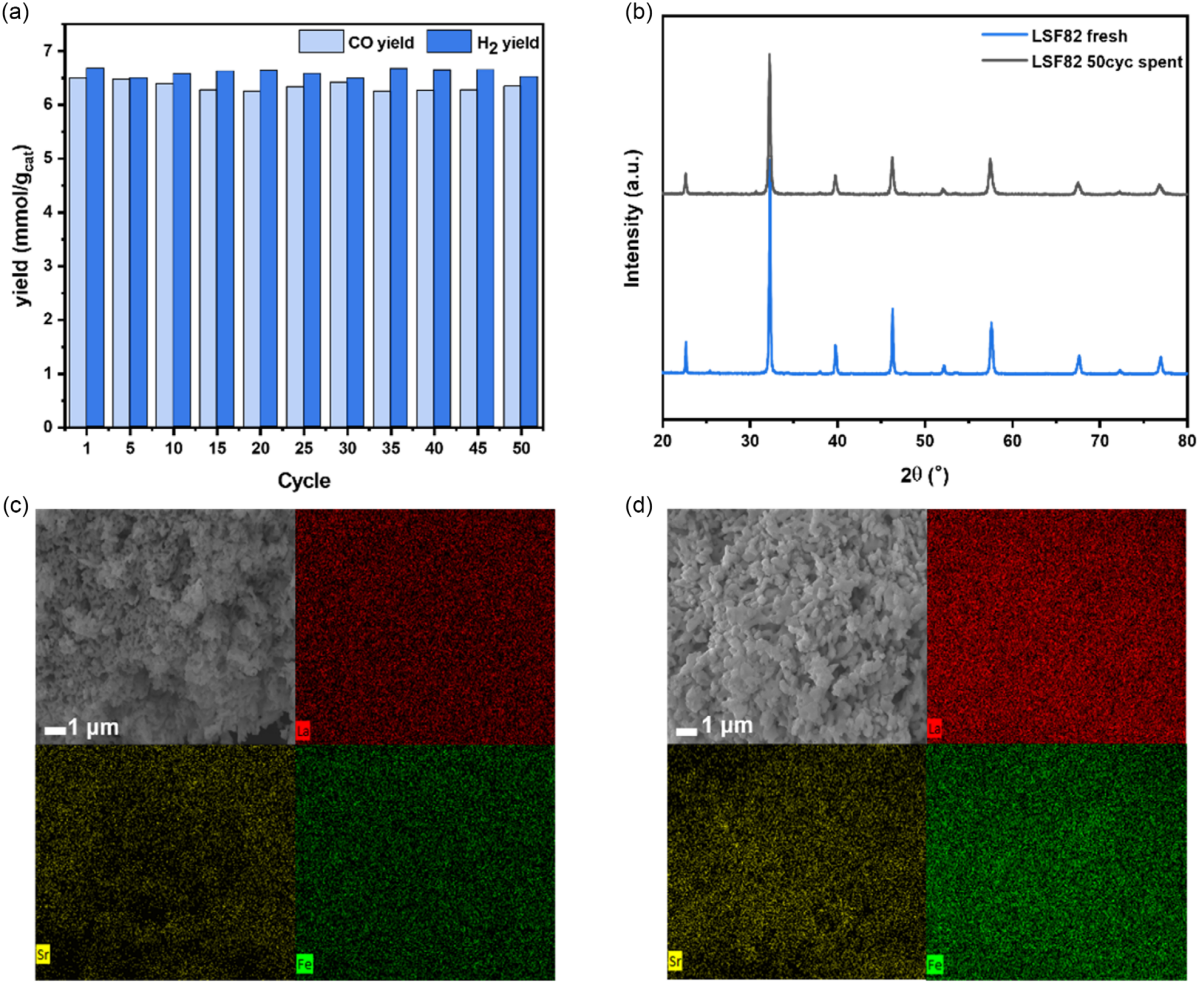
Results for the long‐term stability test of LSF82: a) CO and H_2_ yield of LSF82 during 50‐cycle CL‐SMR, b) XRD patterns of fresh and spent after 50‐cycle CL‐SMR, and SEM and elemental mapping images of c) fresh and d) spent after 50 cycles.

Based on overall performance, including CO yield, coke mitigation, H_2_ yield, and stability, LSF82 was deemed the most promising oxygen carrier and thus selected for further evaluation in a 50‐cycle CL‐SMR test to assess its long‐term durability. As shown in **Figure** **6**a (Figure S14, Supporting Information), the CO yield remained in the range of 6.22–6.51 mmol/g_cat_, and the H_2_ yield from the water splitting step ranged from 6.48 to 6.69 mmol/g_cat_ throughout the 50 cycles. This demonstrates excellent durability without noticeable catalytic deactivation. To investigate potential structural degradation, XRD and scanning electron microscopy‐energy dispersive X‐ray spectroscopy (SEM‐EDS) analyses were conducted after the 50‐cycle test. As shown in Figure 6b, the XRD pattern of LSF82 after 50 CL‐SMR cycles closely matches that of the fresh LSF82 sample. The spent sample after 50‐cycle CL‐SMR retains the orthorhombic perovskite structure observed in the fresh sample and shows no evidence of phase segregation or the formation of secondary phases. In Figure 6c,d, although slight sintering and agglomeration were observed, elemental mapping revealed that all constituent elements remained homogeneously distributed without any elemental segregation. These results indicate that the original orthorhombic perovskite structure was well preserved without noticeable phase segregation or structural deformation during the 50‐cycle CL‐SMR. Thus, LSF82 can produce a large amount of CO and H_2_ over prolonged redox cycles, effectively inhibiting coke formation, which is attributed to facile oxygen transfer. Consequently, the Sr doping at the A‐site of LaFeO_3_ is the most effective approach for enhancing oxygen transfer and thereby improving syngas and hydrogen production while suppressing coke formation in CL‐SMR.

## 3 Conclusion

This study has explored the effect of A‐site doping with alkaline earth metals on the redox properties and catalytic performance of LaFeO_3_‐based perovskite materials in CL‐SMR. A series of orthorhombic perovskites, LaFeO_3_ (LF), La_0.8_Ca_0.2_FeO_3_ (LCF82), La_0.8_Sr_0.2_FeO_3_ (LSF82), and La_0.8_Ba_0.2_FeO_3_ (LBF82), were synthesized and evaluated through cyclic redox experiments to assess their effectiveness in syngas and hydrogen production. Among the samples, LSF82 demonstrated the most favorable performance, exhibiting the highest CO and H_2_ yields alongside the lowest coke deposition. This superior performance is attributed to its enhanced oxygen transfer capability, which results from a substantial increase in the oxygen vacancy concentration. The rapid diffusion of lattice oxygen from the bulk to the surface in LSF82 promotes the selective oxidation of surface‐adsorbed methane into CO and H_2_, inhibiting methane decomposition and coke formation. The suppression of coke deposition during the methane reduction step further enables the production of high‐purity hydrogen during the subsequent water splitting step. In contrast, the undoped LaFeO_3_ (LF) exhibited slower oxygen transfer kinetics, resulting in a higher propensity for methane decomposition and coke accumulation, which adversely affected hydrogen purity. Importantly, LSF82 maintained structural integrity and stable redox performance over 50 cycles, achieving consistent CO and H_2_ yields of 6.22–6.51 mmol/g_cat_ and 6.48–6.69 mmol/g_cat_, respectively. Overall, the results demonstrate that doping Sr at the A‐site of LaFeO_3_ is the most effective strategy to enhance the oxygen transfer property, inhibiting coke deposition and increasing the productivity of syngas and hydrogen in CL‐SMR.

## 4 Experimental Section

4.1

4.1.1

##### Synthesis of Oxygen Carriers

LaFeO_3_ and La_1−*x*_A_*x*_FeO_3_ (*x* = 0.1, 0.2, 0.3; A = Ca, Sr, Ba) were synthesized via a modified Pechini method, as described in previous studies.^[^ ^9^, ^58^ ^]^ Stoichiometric amounts of La(NO_3_)_3_·6H_2_O (Sigma‐Aldrich, 99.99%), Fe(NO_3_)_3_·9H_2_O (Sigma‐Aldrich, >99%), Ca(NO_3_)_2_·4H_2_O (Sigma‐Aldrich, 99%), Sr(NO_3_)_2_ (Sigma‐Aldrich, ≥99.0%), and Ba(NO_3_)_2_ (Sigma‐Aldrich, ≥99%) were dissolved in 50 mL of deionized water and stirred at 50 °C for 30 min at 500 rpm. Citric acid anhydrous (CA, ≥99.5%, Junsei) was added at a molar ratio of 3:1 for the total metal cations, followed by further stirring at 50 °C for 30 min until complete dissolution was achieved. Upon cooling to room temperature, aqueous NH_4_OH (Samchun, 25–30%) was added dropwise while stirring at 300 rpm until the pH reached 7, as monitored by a pH meter (Mettler Toledo). The pH was adjusted to 7 following our previous findings, which demonstrated that LaFeO_3_ synthesized at pH 7 exhibited the most homogeneous chelation and highest performance in CL‐SMR.^[^ ^9^ ^]^

To facilitate esterification and enhance chelation, ethylene glycol (EG, ≥99.5%, Samchun) was added dropwise at a molar ratio of 2:1 relative to citric acid. The solution was stirred at 80 °C for at least 3 h at 500 rpm, followed by drying overnight at 100 °C to form a polymeric resin. Further drying was performed at 180 °C to ensure complete removal of moisture. The dried resin was then calcined in an alumina crucible: first at 450 °C for 4 h to remove volatile species and subsequently at 900 °C for 8 h to eliminate organic residues. A ramping rate of 5 °C min^−1^ was used under an air flow of 80 mL min^−1^. The final powders were ground using a mortar and pestle. The undoped‐LaFeO_3_ sample was labeled as LF. The A‐site doped samples were denoted as L*A*F*xy*, where “A” represents the dopant cation (C for Ca, S for Sr, and B for Ba), and *x* and *y* correspond to the La and dopant molar ratios, respectively (with *x* = 10 − *y*, and *y* = 1, 2, 3 for 10%, 20%, and 30% doping, respectively).

##### Characterization of Oxygen Carriers

A synchrotron XRD analysis was conducted at the 9B HRPD (high‐resolution powder diffraction) beamline of PLS‐II (Pohang Light Source, Pohang Accelerator Laboratory, Korea) to investigate the crystal structure and lattice parameter variations. Monochromatic X‐rays with a wavelength of 1.546 Å were used, and diffraction patterns were collected over the 2*θ* range of 20° to 80° with a step size of 0.02°. The acquired data were refined using the Rietveld method implemented in FullProf software. TEM (FEI Talos F200X, KAIST Analysis Center for Research Advancement) was used to analyze the elemental distribution and interplanar spacing (d‐spacing) of the samples. HAADF‐STEM provided both dark‐field images and elemental mapping. High‐resolution TEM (HR‐TEM) images were processed using DigMicrograph software (GATAN). Elemental compositions were determined by ICP‐OES (Agilent 720, KAIST Analysis Center for Research Advancement). Surface chemical states and species were identified via X‐ray photoelectron spectroscopy (XPS, Thermo VG Scientific, K‐alpha, KAIST Analysis Center for Research Advancement) using Al Kα radiation (1486.7 eV). All spectra were calibrated against the C *1s* peak at 284.8 eV and analyzed using the Thermo Avantage software. Surface morphology and structural integrity before and after redox cycling were examined through field‐emission scanning electron microscopy (FE‐SEM, Jeol JSM‐IT800, KAIST Analysis Center for Research Advancement).

The oxygen content of the synthesized oxygen carriers was determined by cerimetric titration. A blank solution was prepared by mixing 5 mL of 0.1 N Mohr's salt solution with 5 mL of 6 M HCl, without adding any sample. A 100 mg sample was added to a 0.1 N Mohr's salt solution, followed by the addition of 5 mL of 6 M HCl. The mixture was then heated at 90 °C for 30 min to ensure complete dissolution of the sample. After cooling the solution to room temperature, a drop of ferroin solution (Daejung) indicator was added. While stirring, a cerium(IV) sulfate 0.1 N standardized solution (ThermoFisher Scientific) was added dropwise until the endpoint was visually observed through a change of color from orange‐yellow to light green. The volume of cerium sulfate solution required to reach the endpoint was used to calculate the Fe valence state and the corresponding oxygen content in the sample. The detailed calculation is demonstrated in the ‘Oxygen content calculation section’ in the Supporting Information.

CH_4_‐temperature programmed surface reaction (CH_4_‐TPSR) was performed on fresh samples by loading 80 mg of material into a fixed‐bed quartz reactor (inner diameter: 7 mm). The sample was heated to 900 °C at a ramping rate of 5 °C min^−1^ under 5 mL min^−1^ CH_4_ balanced with 45 mL min^−1^ Ar. Reaction products were analyzed using a mass spectrometer (MS, QMG250, Pfeiffer Vacuum). O_2_‐temperature programmed desorption (O_2_‐TPD) was also conducted on fresh and spent samples. First, 100 mg of sample was pretreated at 550 °C for 1 h under a flow of 5 mL min^−1^ O_2_ and 45 mL min^−1^ Ar. After cooling to room temperature, 50 mL min^−1^ Ar was flowed for 30 min to remove residual O_2_. The sample was then heated to 900 °C at 5 °C min^−1^ under an Ar flow, and the desorbed O_2_ was monitored by MS.

##### CL‐SMR Experiments

CL‐SMR experiments were carried out in a fixed‐bed quartz reactor. First, 0.15 g of oxygen carrier was loaded and heated to 800 °C at a ramping rate of 5 °C min^−1^ using an electric furnace. Each CL‐SMR cycle consisted of three sequential steps. In the reduction step, the oxygen carrier was exposed to 5 mL min^−1^ of CH_4_ balanced with 45 mL min^−1^ of Ar for 10 min. In the subsequent water‐splitting step, deionized water was delivered at 0.005 mL min^−1^ via a syringe pump, vaporized using a steam generator, and introduced into the reactor for 15 min, with Ar as the carrier gas (45 mL min^−1^). In the final combustion step, the oxygen carrier was reoxidized by supplying 5 mL min^−1^ of O_2_ balanced with 45 mL min^−1^ of Ar for 5 min. Argon (50 mL min^−1^) was used to purge the system for 10 min between each step to prevent gas mixing. The three‐step sequence was repeated for 10 cycles to assess the redox reactivity and extended to 50 cycles to evaluate the long‐term stability. All effluent gases were continuously monitored using MS.

## Supporting Information

The authors have cited additional references within the Supporting Information.^[^ ^21^, ^59^, ^60^ ^]^

## Conflict of Interest

The authors declare no conflict of interest.

## Supporting information

Supplementary Material

## Data Availability

The data that support the findings of this study are available from the corresponding author upon reasonable request.
