# Supplementary material for: Modulating Oxygen Transfer via A‐Site Doping in LaFeO3 for Coke‐Resistant Chemical Looping Steam Methane Reforming
Source: ChemSusChem. 2025 Oct 8;18(24):e202501667. doi: 10.1002/cssc.202501667 (PMC12703423; doi:10.1002/cssc.202501667)
Supplement: Supplementary file 1 — Supplementary Material [file CSSC-18-e202501667-s001.pdf]

Supporting Information

Modulating Oxygen Transfer via A-Site Doping in $LaFeO_3$ for Coke-Resistant Chemical Looping Steam Methane Reforming

Jeongin Ha, Hyeon Seok Kim, Hyunjung Kim, Yikyeom Kim, Surya Ayuati Ning Asih and Jae W. Lee*

Department of Chemical and Biomolecular Engineering, Korea Advanced Institute of Science and Technology (KAIST), 291 Daehak-Ro, Daejeon 34141, Republic of Korea

* Corresponding author: e-mail: jaewlee@kaist.ac.kr; Tel: +82-42-350-3940; FAX: +82-42-350-3910

1. Extended experimental section

1.1 Determination of optimal doping ratio

CL-SMR experiments were conducted for LCFxy, LSFxy, and LBFxy (x = 10-y, y = 1, 2, 3) to determine the optimal doping ratio. 0.15 g of the oxygen carrier was loaded into the fixed-bed quartz reactor and heated to 750 °C at a rate of 5 °C/min using the electric furnace. During the reduction step, the oxygen carrier was reduced with 5 mL/min of CH_4 balanced with 45 mL/min of Ar for 5 minutes. For the water splitting step, deionized water was pumped at a rate of 0.005 mL/min and injected into the reactor for 10 minutes, carried by 45 mL/min of Ar. For the combustion (regeneration) step, 5 mL/min of O_2 balanced with 45 mL/min of Ar was supplied for 5 minutes. Between each step, the system was purged with 50 mL/min of Ar for 10 minutes. All effluent gases were continuously detected and monitored by MS.

1.2 Determination of operating temperature

To determine the optimal operating temperature for CL-SMR, experiments were carried out using LSF82 at 700 °C, 750 °C, and 800 °C. A fixed-bed quartz reactor was loaded with 0.15 g of the oxygen carrier and heated to the target temperature at a rate of 5 °C/min using an electric furnace. The reduction step was performed by introducing 5 mL/min of CH_4 (balanced with 45 mL/min of Ar) for 5 minutes. During the subsequent water-splitting step, deionized water was fed at 0.005 mL/min and carried into the reactor by 45 mL/min of Ar for 10 minutes. In the combustion step, 5 mL/min of O_2 (also balanced with 45 mL/min of Ar) was supplied for 5 minutes. 50 mL/min of Ar was used to purge the system for 10 minutes between each step. The outlet gas composition was continuously monitored using MS.

2. Oxygen content calculation section

The amount of Fe^{2+} from the perovskite sample can be determined by subtracting the volume of cerium sulfate solution used for titrating the blank solution from that used for the sample. A positive value indicates the presence of Fe^{2+}, whereas a negative value suggests the presence of Fe^{4+} in the sample.

$$(mol\ of\ Fe^{2+}\ from\ sample) = (mol\ of\ cerium\ sulfate\ solution\ for\ sample\ titration) - (mol\ of\ cerium\ sulfate\ solution\ for\ blank\ solution\ titration)$$

The molar amount of oxygen is calculated based on the total cationic charge of the sample.

$$(mol\ of\ A-site\ cation) = \{3 \times (atomic\ ratio\ of\ La^{3+}) + 2 \times (atomic\ ratio\ of\ alkaline\ earth\ metal\ cation)\} \times (mol\ of\ sample)$$

$$(mol\ of\ B-site\ cation) = 3 \times (mol\ of\ sample) - (mol\ of\ Fe^{2+}\ from\ sample)$$

$$(mol\ of\ oxygen) = \frac{(mol\ of\ A-site\ cation) + (mol\ of\ B-site\ cation)}{2}$$

The oxygen non-stoichiometry (3-δ) of the perovskite, based on the general formula $ABO_{3-\delta}$, is calculated as follows.

$$(3-\delta) = \frac{(mol\ of\ oxygen)}{(mol\ of\ sample)}$$

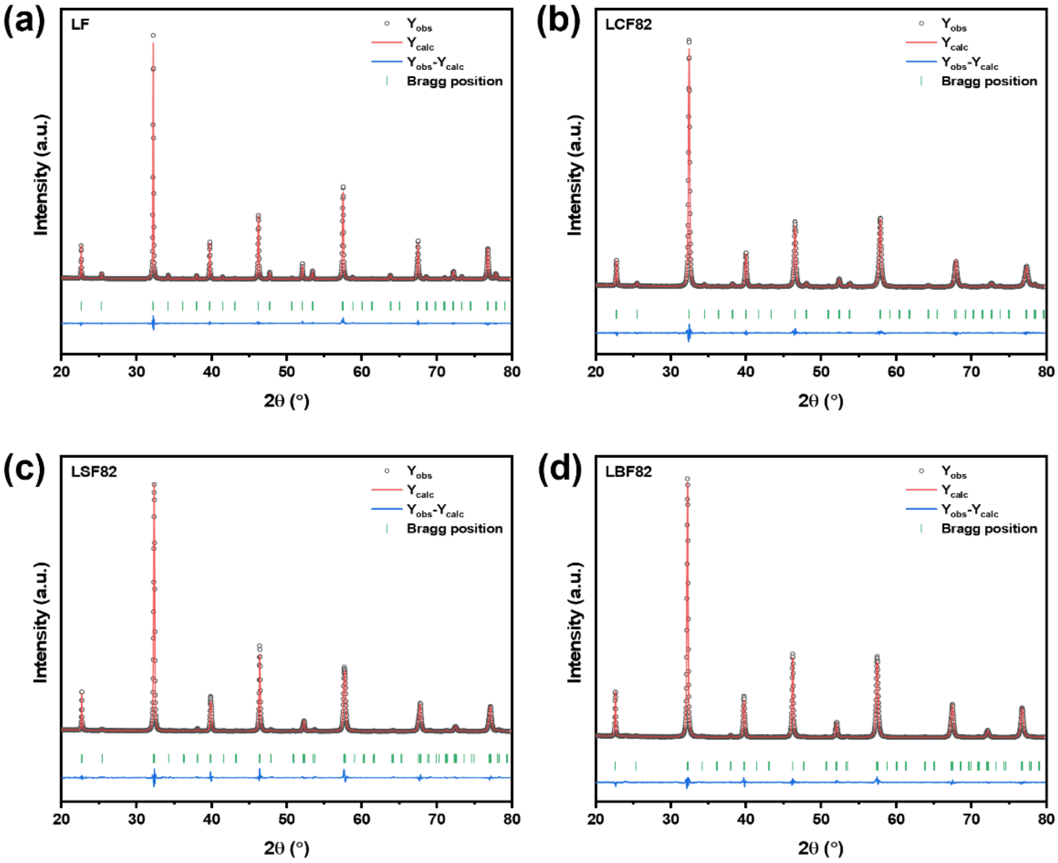

Figure S1. HRPD Rietveld fitting of fresh oxygen carriers: (a) LF, (b) LCF82, (c) LSF82, and (d) LBF82.

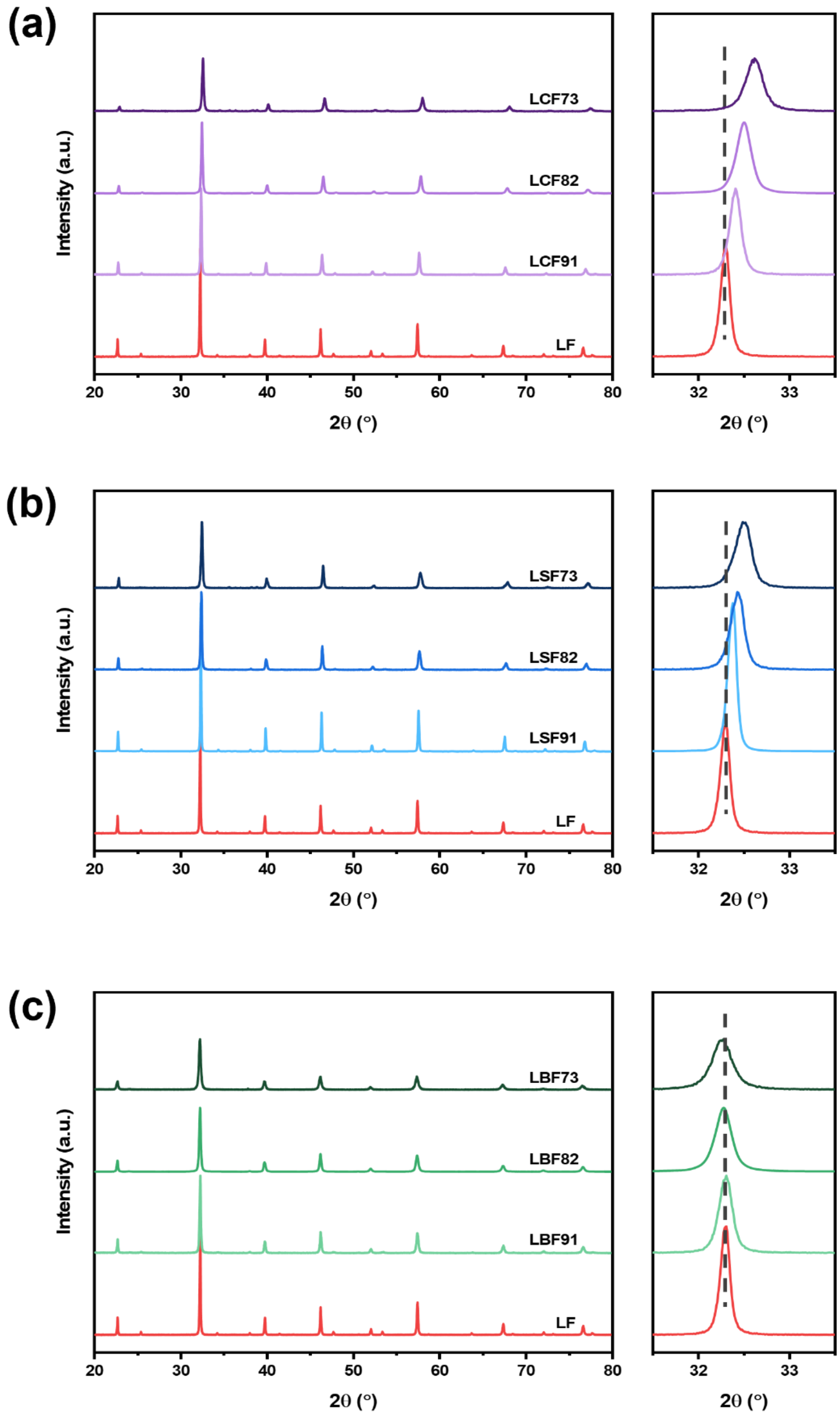

Figure S2. XRD patterns of (a) LF, LCF91, LCF82, and LCF73; (b) LF, LSF91, LSF82, and LSF73; and (c) LF, LBF91, LBF82, and LBF73.

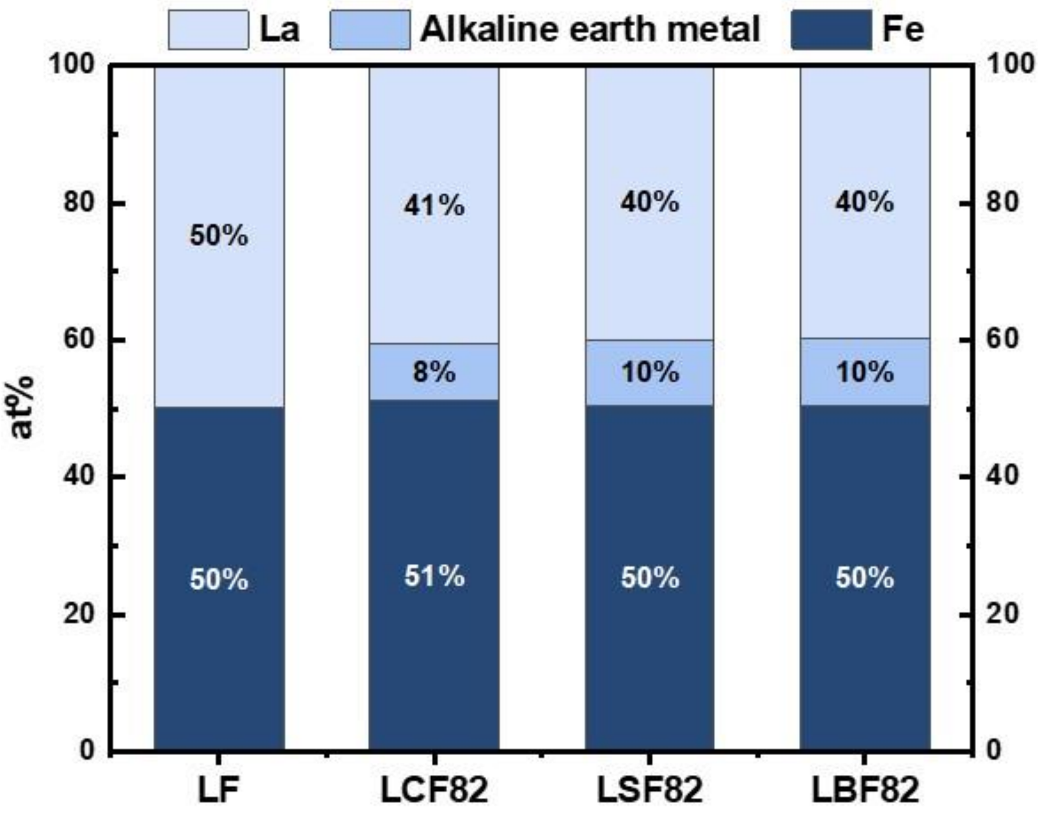

Figure S3. Metal ratio of synthesized LF, LCF82, LSF82, and LBF82 determined by ICP-OES.

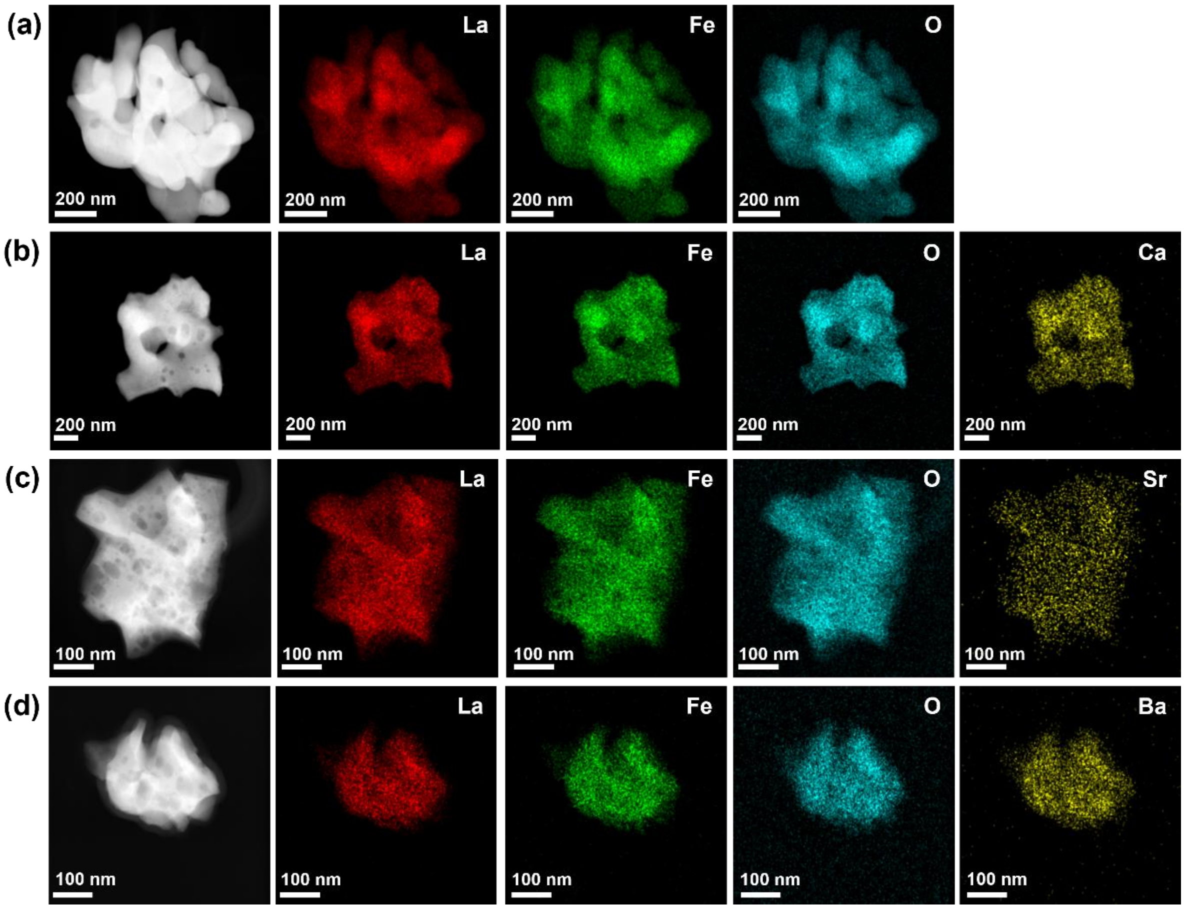

Figure S4. HAADF-STEM and element mapping images of (a) LF, (b) LCF82, (c) LSF82, and (d) LBF82.

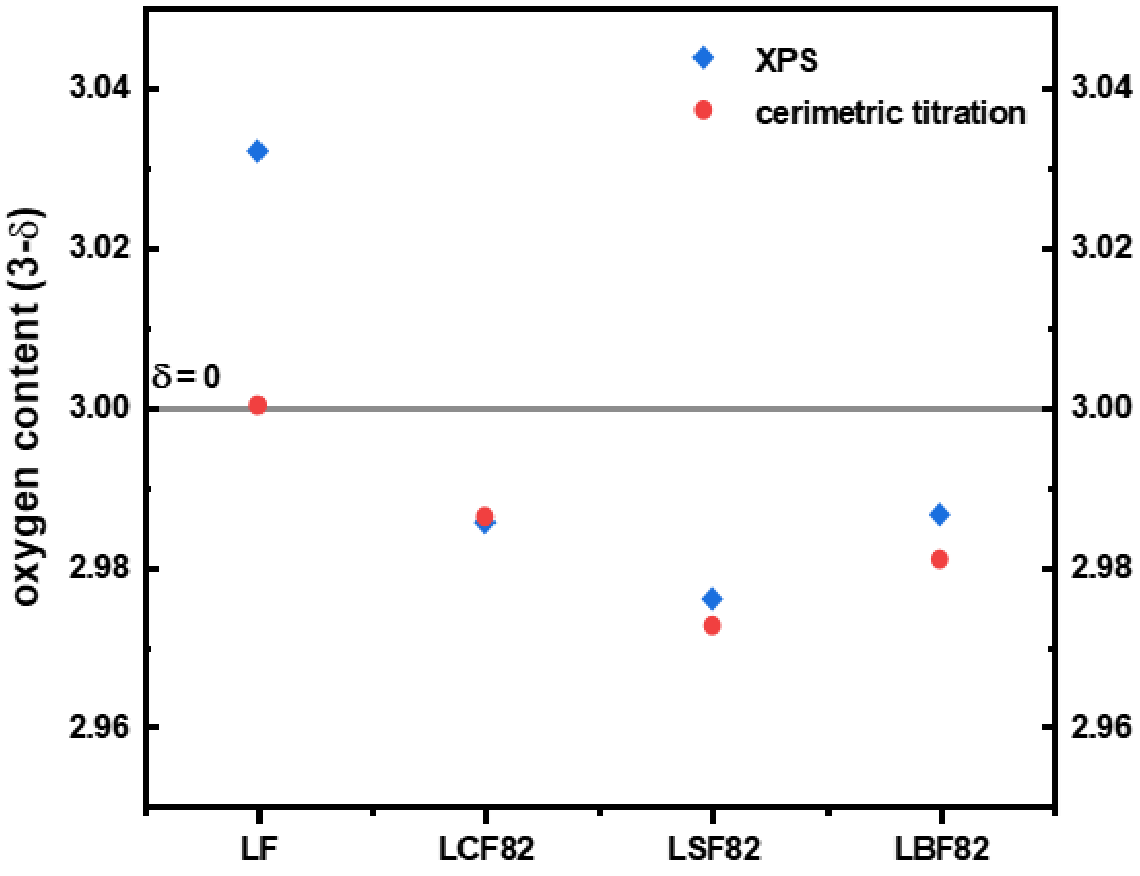

Figure S5. Oxygen content determined by Fe *2p* XPS and cerimetric titration.

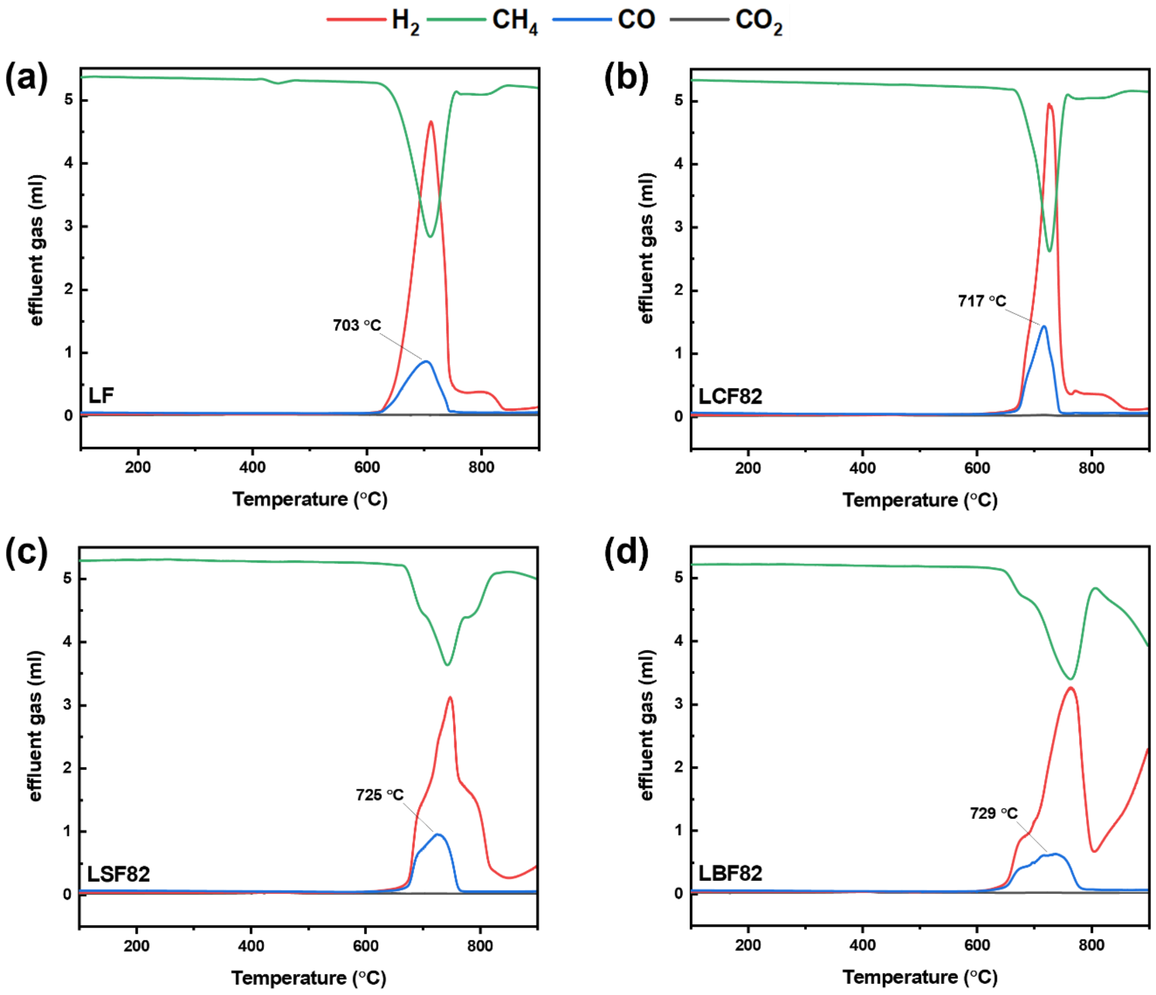

Figure S6. Real-time profile of effluent gas during CH_4-TPSR for (a) LF, (b) LCF82, (c) LSF82, and (d) LBF82.

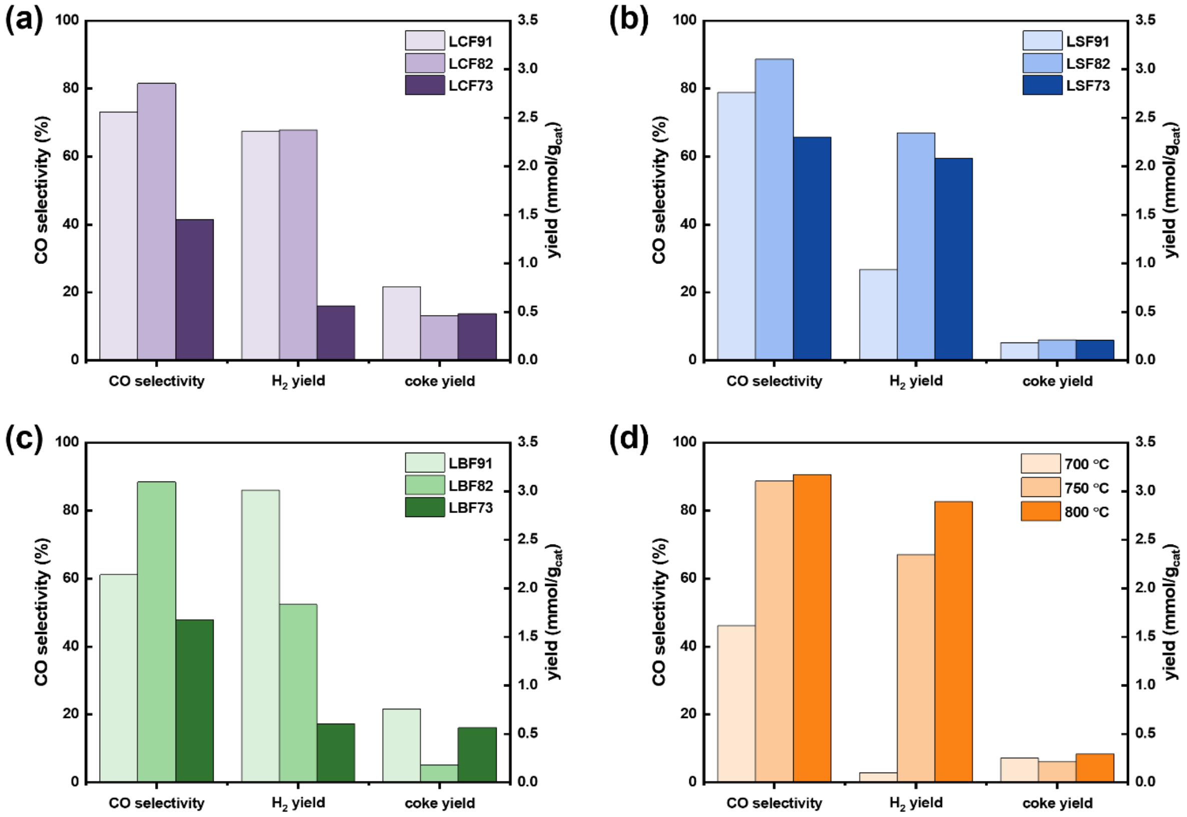

Figure S7. CL-SMR performance of oxygen carriers at different doping ratios of (a) LCFxy, (b) LSFxy, and (c) LBFxy, (where x=10-y, y=1, 2, 3); and (d) different reaction temperatures, showing CO selectivity, coke yield during the reduction step, and hydrogen yield during the water splitting step.

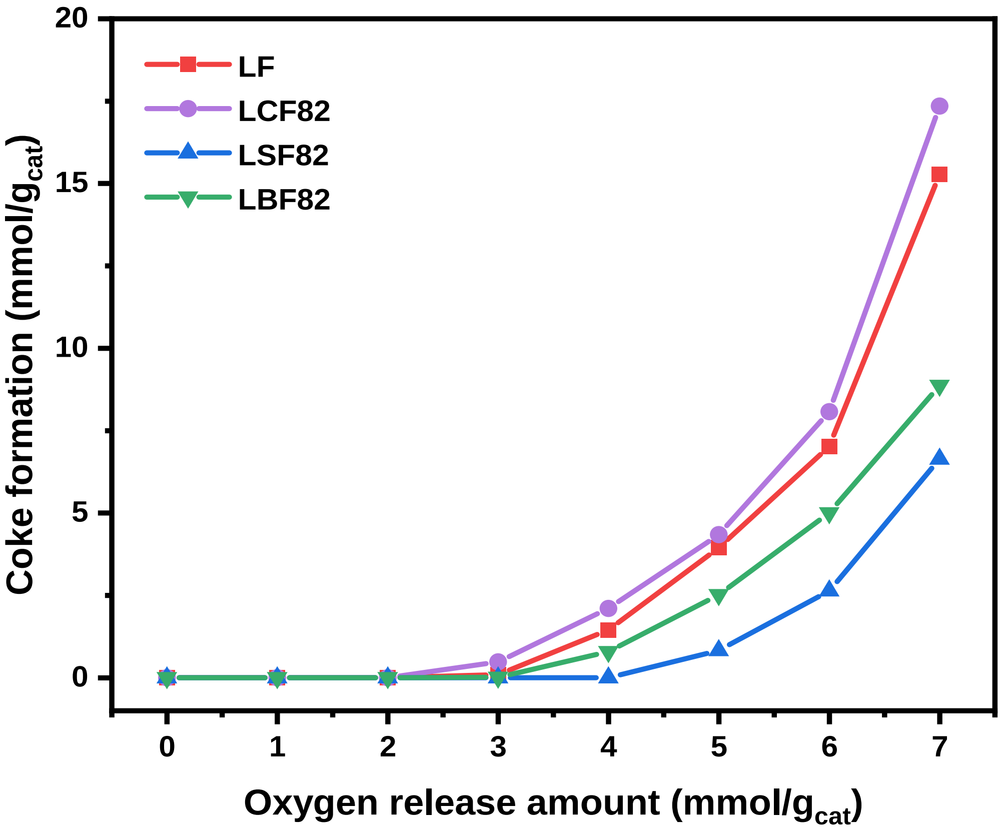

Figure S8. Coke formation dependent on oxygen release during methane reduction (800 °C, 30 min).

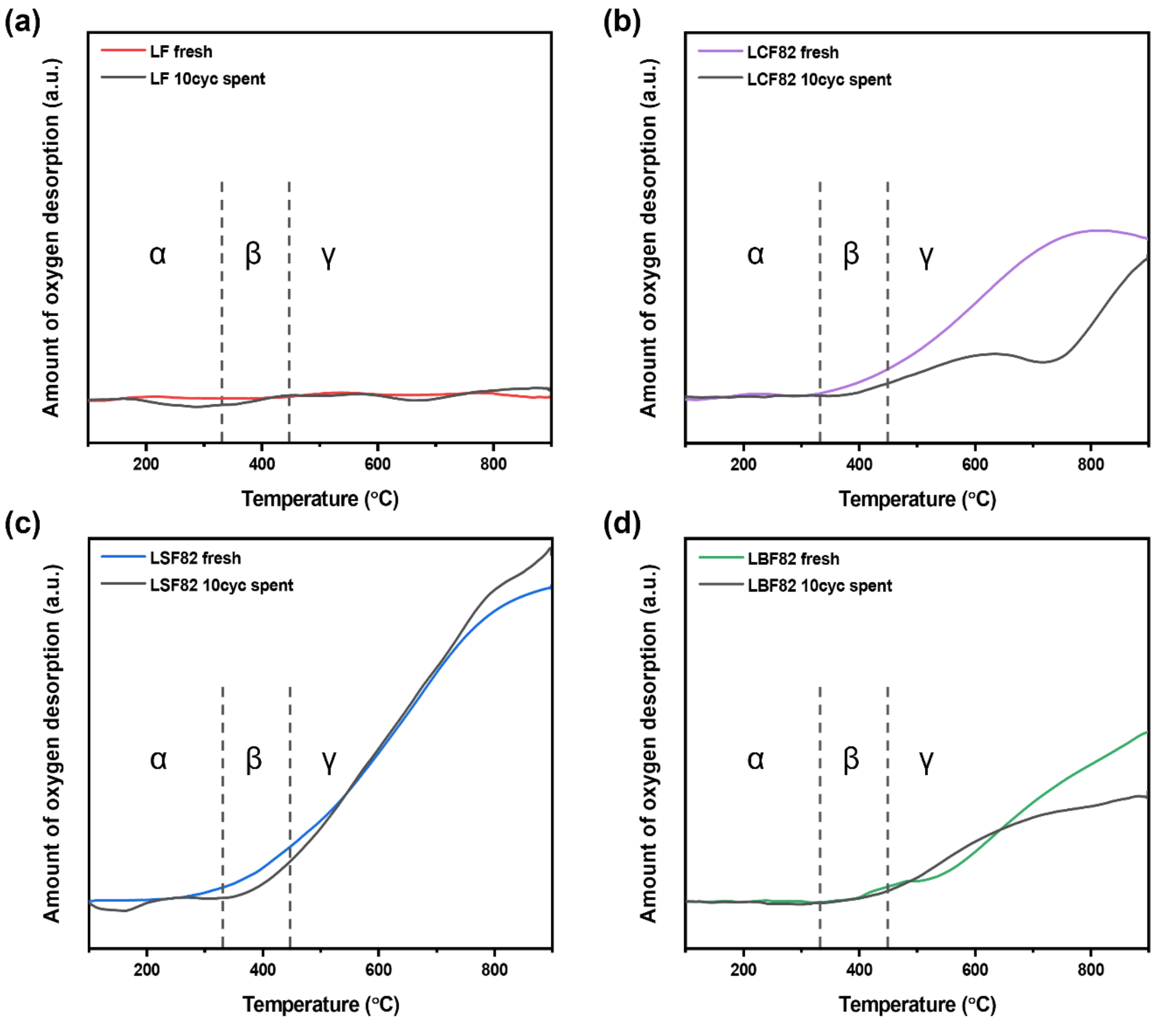

Figure S9. O_2-TPD of fresh and 10 cycles spent samples: (a) LF, (b) LCF82, (c) LSF82, and (d) LBF82.

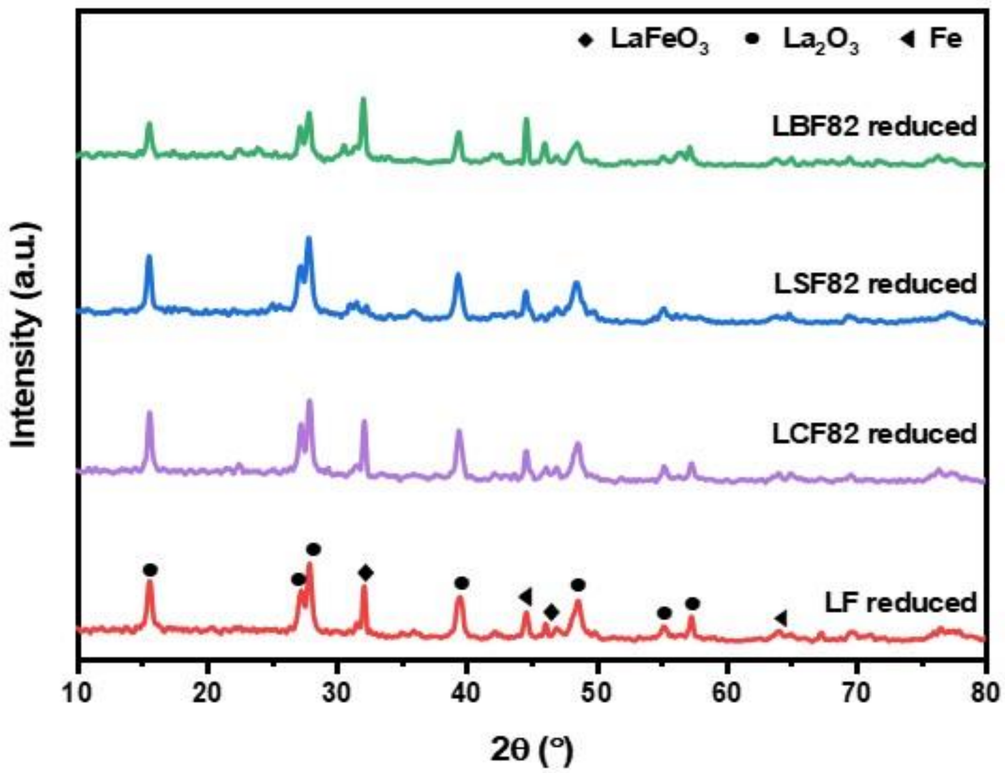

Figure S10. XRD patterns of samples reduced in methane (800 °C, 10 min).

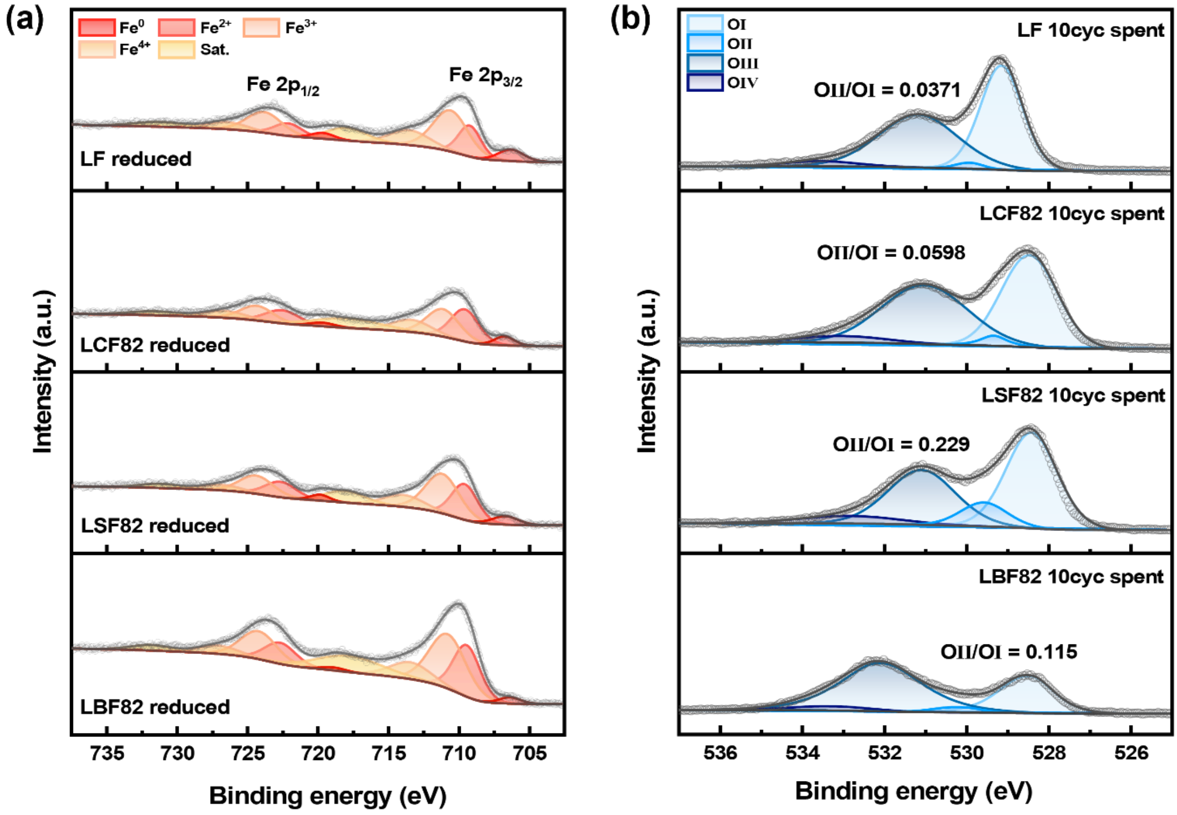

Figure S11. (a) Fe *2p* XPS spectra for samples reduced in methane (800 °C, 10 min), and (b) O *1s* XPS spectra for spent samples after 10 CL-SMR cycles.

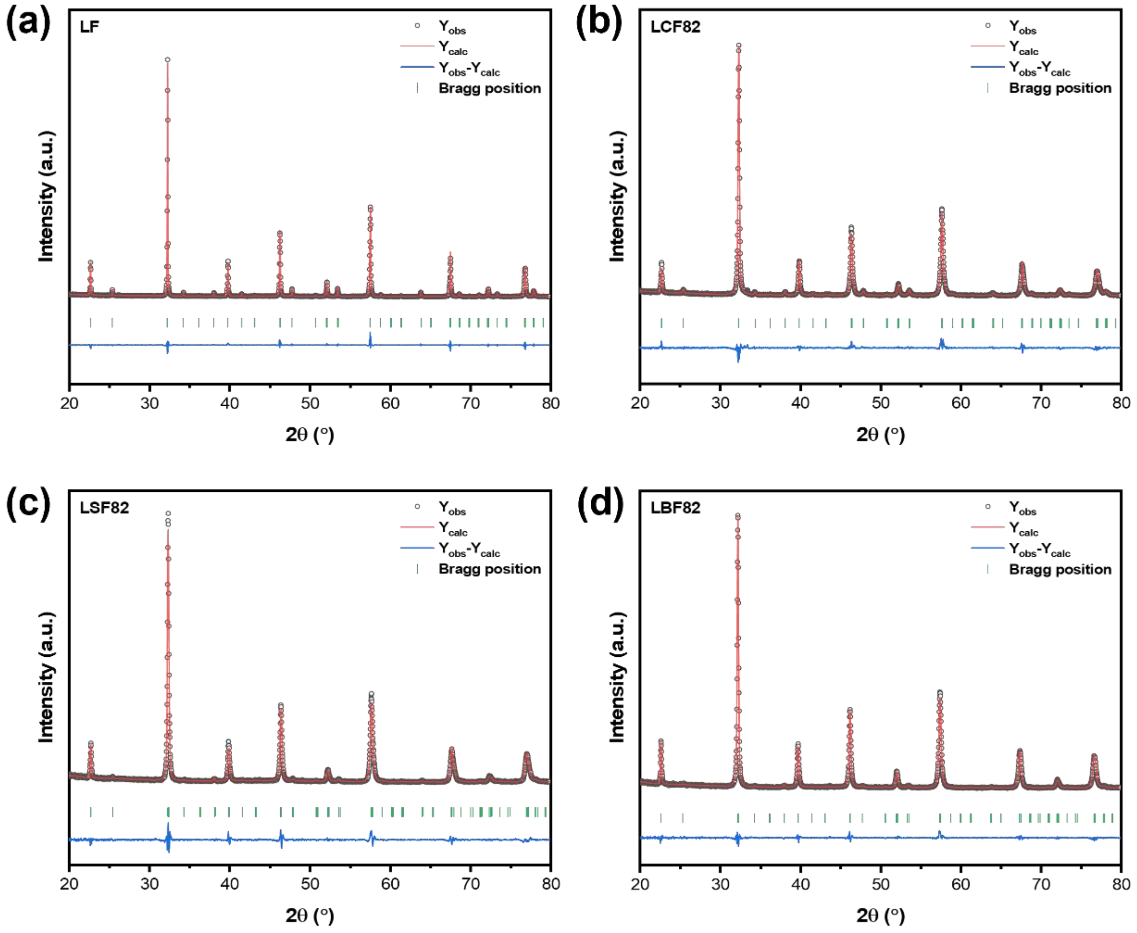

Figure S12. XRD fitting results of the spent oxygen carriers after 10 CL-SMR cycles: (a) LF, (b) LCF82, (c) LSF82, and (d) LBF82

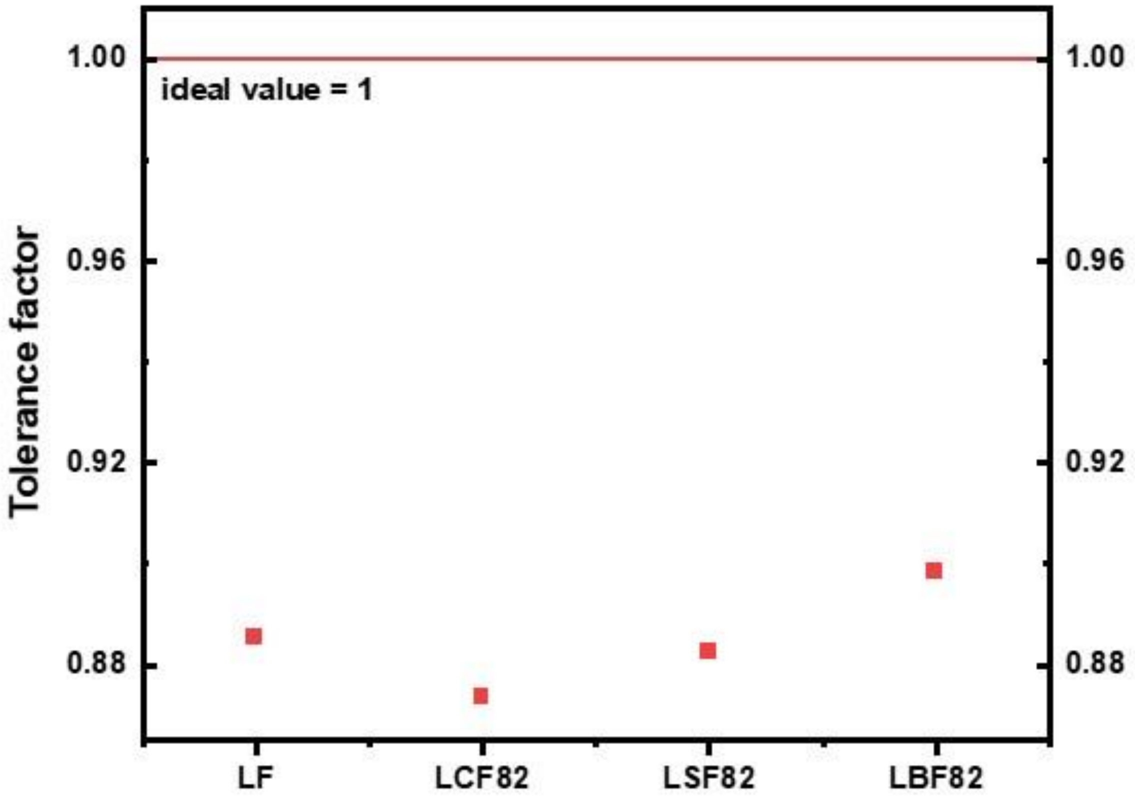

Figure S13. Goldschmidt tolerance factor of LF, LCF82, LSF82, and LBF82.

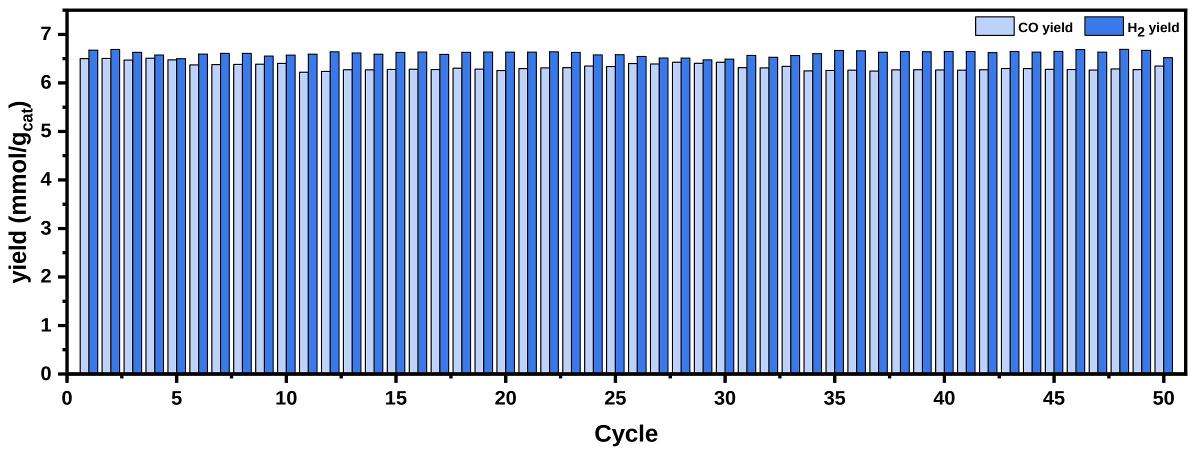

Figure S14. CL-SMR performance of LSF82 during 50 cycles.

Table S1. O_2 release amount and oxygen storage capacity.

	O_2 release amount (mmol O_2/g_{cat})[a]	Experimental oxygen storage capacity (mmol O_2/g_{cat})[b]	Theoretical oxygen storage capacity (mmol O_2/g_{cat})	Extracted oxygen (%)
LF	0.0151	4.88	6.18	79.0
LCF82	0.224	5.27	6.73	78.3
LSF82	0.377	5.72	6.45	88.7
LBF82	0.158	5.20	6.19	84.0

[a] calculated values from O_2-TPD. [b] Calculated values from CH_4-TPSR.

Table S2. Comparison of oxygen storage capacity (OSC).

	Oxygen storage capacity (mmol O_2/g_{cat})	Reference
$LaCoO_3$	~2.5	[59]
$LaMn_{0.9}Ni_{0.1}O_3$	~0.8	[60]
$La_{0.5}Sr_{0.5}CoO_3$	~3.0	[21]
LSF82	5.72	This study

Table S3. Atomic ratio of iron species for samples reduced in methane (800 °C, 10 min) determined by Fe *2p* XPS.

at %	Fe^0	Fe^{2+}	Fe^{3+}	Fe^{4+}
LF	9.43	23.17	47.65	19.75
LCF82	7.06	35.56	36.79	20.61
LSF82	7.55	31.97	44.41	16.07
LBF82	3.30	32.60	47.96	16.14

Table S4. Refinement results of HRPD experiment for spent perovskites.

Spent sample	Lattice parameter				Cell volume (Å^3)	Volume change (V_{spent}-V_{fresh}, Å^3)
	a (Å)	b (Å)	c (Å)	α=β=γ (°)		
LF	5.565	7.855	5.556	90	242.90	0.05
LCF82	5.547	7.831	5.554	90	241.28	2.78
LSF82	5.551	7.851	5.527	90	240.88	0.56
LBF82	5.556	7.880	5.573	90	244.01	0.96

Table S5. Atomic ratio of oxygen species and OII/OI determined by O *1s* XPS for spent samples after 10 CL-SMR cycle.

at%	OI (O^{2-})	OII (O_2^{2-}, O-)	OIII (CO_3^{2-}, OH^-)	OIV (H_2O)	OII/OI
LF	48.22	1.79	45.83	4.17	0.0371
LCF82	45.97	2.75	45.58	5.70	0.0598
LSF82	46.79	10.72	35.25	7.24	0.229
LBF82	30.79	3.53	60.60	5.08	0.115
